# Ferroelectric Order Evolution in Freestanding PbTiO_3_ Films Monitored by Optical Second Harmonic Generation

**DOI:** 10.1002/advs.202307571

**Published:** 2024-06-24

**Authors:** Sisi Huang, Shuai Xu, Cheng Ma, Pengzhan Li, Er‐Jia Guo, Chen Ge, Can Wang, Xiulai Xu, Meng He, Guozhen Yang, Kuijuan Jin

**Affiliations:** ^1^ Beijing National Laboratory for Condensed Matter Physics Institute of Physics Chinese Academy of Sciences Beijing 100190 China; ^2^ University of Chinese Academy of Sciences Beijing 100049 China; ^3^ Songshan Lake Materials Laboratory Dongguan Guangdong 523808 China; ^4^ State Key Laboratory for Mesoscopic Physics and Frontiers Science Center for Nano‐optoelectronics School of Physics Peking University Beijing 100871 China

**Keywords:** curie temperature, ferroelectric order, freestanding PbTiO_3_ films, second harmonic generation, strain

## Abstract

The demand for low‐dimensional ferroelectric devices is steadily increasing, however, the thick substrates in epitaxial films impede further size miniaturization. Freestanding films offer a potential solution by eliminating substrate constraints. Nevertheless, it remains an ongoing challenge to improve the stability in thin and fragile freestanding films under strain and temperature. In this work, the structure and ferroelectric order of freestanding PbTiO_3_ (PTO) films are investigated under continuous variation of the strain and temperature using nondestructive optical second harmonic generation (SHG) technique. The findings reveal that there are both out‐of‐plane and in‐plane domains with polarization along out‐of‐plane and in‐plane directions in the orthorhombic‐like freestanding PTO films, respectively. In contrast, only out‐of‐plane domains are observed in the tetragonal epitaxial PTO films. Remarkably, the ferroelectricity of freestanding PTO films is strengthened under small uniaxial tensile strain from 0% up to 1.66% and well‐maintained under larger biaxial tensile strain up to 2.76% along the [100] direction and up to 4.46% along the [010] direction. Moreover, a high Curie temperature of 630 K is identified in 50 nm thick freestanding PTO films by wide‐temperature‐range SHG. These findings provide valuable understanding for the development of the next‐generation electronic nanodevices with flexibility and thermostability.

## Graphical abstract

In this work, freestanding PbTiO_3_ films exhibit an orthorhombic‐like phase, whereas the tetragonal phase is observed in epitaxial PbTiO_3_ films. Remarkably, the ferroelectricity is strengthened under small uniaxial tensile strain up to 1.66% in the 50 nm‐thick freestanding PbTiO_3_ films with Curie temperature of 630 K. These findings provide valuable understanding for fabricating electronic nanodevices with flexibility and thermostability.

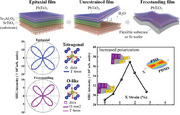

## 1 Introduction

Ferroelectric materials have attracted intense interest due to their extensive applications in memory devices, sensors, and capacitors.^[^ ^1^, ^2^, ^3^, ^4^ ^]^ As the trend toward miniaturizing ferroelectric devices continues, the demand for low‐dimensional nanomaterials with excellent ferroelectricity is increasing. Growing nanoscale‐thick epitaxial films on single‐crystal oxide substrates has been a traditional approach to reduce dimensions and precisely engineer the properties at atomic level.^[^ ^5^, ^6^, ^7^, ^8^, ^9^, ^10^, ^11^, ^12^ ^]^ However, epitaxial films have inherent limitations, being fixed with thick substrates, which hinders the realization of isolated two‐dimensional layers.^[^ ^13^, ^14^, ^15^, ^16^, ^17^, ^18^, ^19^, ^20^ ^]^

Recently, freestanding perovskite oxide films have emerged as a promising solution to overcome the limitations from substrates.^[^ ^21^, ^22^, ^23^, ^24^, ^25^ ^]^ These freestanding films not only preserve high‐quality single‐crystal characteristics in low‐dimension but also can be readily stacked and integrated with dissimilar materials through van der Waals interactions, rendering them compatible with existing technological platforms such as industrial silicon substrates or flexible layers.^[^ ^26^, ^27^, ^28^ ^]^ For instance, researchers have reported the realization of room‐temperature skyrmion‐like polar nanodomains in lead titanate/strontium titanate freestanding bilayers and superlattices, demonstrating the potential of freestanding films for silicon‐based high‐density non‐volatile memory applications.^[^ ^28^, ^29^, ^30^, ^31^, ^32^, ^33^, ^34^, ^35^, ^36^, ^37^, ^38^ ^]^ Moreover, eliminating mechanical boundary constraints from substrates leads to the variation of the physical properties for freestanding films. Enhanced piezoelectricity, for example, has been observed in freestanding PbTiO_3_ (PTO) films via fast cooling, resulting from the presence of domains with polarization along both out‐of‐plane/in‐plane directions (*c*/*a* domains) in freestanding films in contrast to the epitaxial PTO films with only *c* domains.^[^ ^39^ ^]^ Furthermore, freestanding films on flexible layers possess mechanical flexibility, allowing them to be stretched or bent by applying continuous strain.^[^ ^40^, ^41^, ^42^, ^43^ ^]^ Notably, it has been reported that uniaxial tensile strain up to 6.4% can be applied on freestanding PTO films with a thickness of 15 unit cells. However, the inherent fragility of freestanding films leads to the reduced *c*/*a* ratio and the weakened ferroelectricity.^[^ ^40^ ^]^ Consequently, enhancing the ferroelectric properties and the stability of freestanding films remains an ongoing challenge. Moreover, analyzing their robustness against external factors such as mechanical deformation and temperature fluctuations is crucial for ensuring the long‐term performance of freestanding films.

In this work, we systematically investigated the evolution of ferroelectric order in freestanding PTO films using techniques including X‐ray diffraction (XRD), reciprocal space mapping (RSM), piezoresponse force microscope (PFM), and optical second harmonic generation (SHG) measurements, with continuously varying strain and temperature. SHG involves a process where the frequency of incident light is doubled by a material with local inversion symmetry breaking. This process serves as a sensitive and nondestructive probe for characterizing ferroelectric order as the intensity of SHG is proportional to the square of the light‐induced nonlinear polarization and the anisotropy of SHG provides valuable insights about crystal structures.^[^ ^44^, ^45^ ^]^ Our results revealed that the 50 nm‐thick epitaxial PbTiO_3_/Sr_3_Al_2_O_6_/SrTiO_3_ (PTO/SAO/STO) films exhibited a tetragonal phase with only out‐of‐plane domains. By contrast, the freestanding PTO films, which have been aspirated from the STO substrate by dissolving the sacrificed layer SAO, displayed an orthorhombic‐like phase with both out‐of‐plane and in‐plane domains. Both two types of films exhibited robust ferroelectricity. Furthermore, our SHG measurements indicated that the polarization of freestanding PTO films was initially strengthened with small uniaxial tensile strain from 0% up to 1.66%. Besides, even under the application of large biaxial in‐plane tensile strain up to 2.76% along the [100] direction and up to 4.46% along the [010] direction, the ferroelectricity of freestanding PTO films was still well‐preserved. Additionally, through an analysis of both SHG and XRD signals as a function of temperature, we extract a high Curie temperature (a transition temperature from ferroelectric to paraelectric phase,^[^ ^46^ ^]^ *Tc*) of ≈630 K in the 50 nm‐thick freestanding PTO films. Overall, the present study provides valuable insights into the evolution of structure and ferroelectric order of freestanding films subjected to varying tensile strain and temperature. These insights hold significant implications for the development of the next‐generation electronic nanodevices with flexibility and thermostability.

## 2 Results and Discussion

### 2.1 Preparation and Structure Analysis

**Figure 1:**
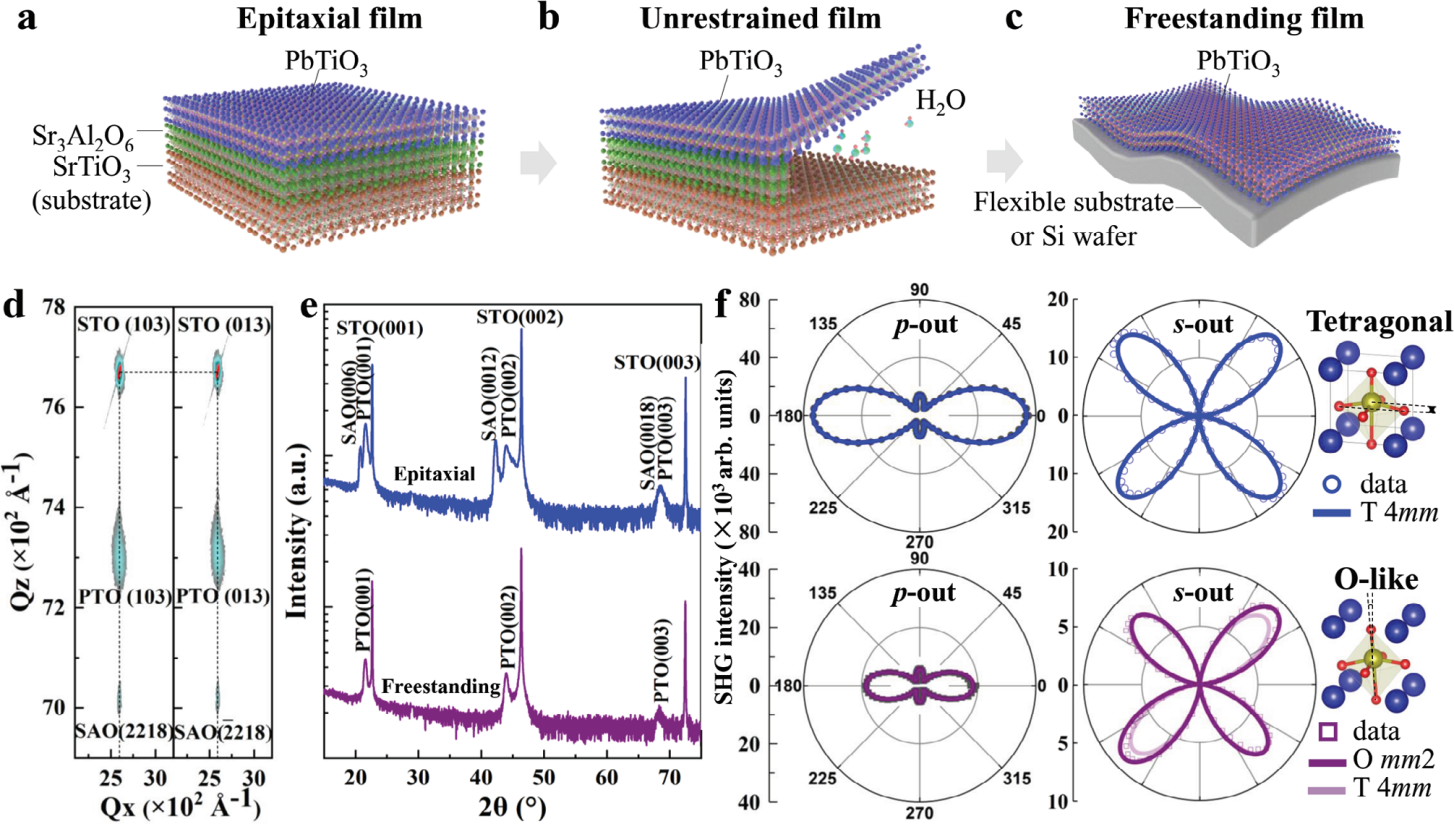
Preparation of freestanding films and structure comparison between epitaxial PTO/SAO/STO films and freestanding PTO films. a‐c) Schematic illustration of the lift‐off process, where the 60 nm‐thick SAO water‐soluble sacrificed layer is first grown on STO (001) substrates followed with the 50 nm‐thick ferroelectric PTO layer. After dissolving SAO by water, freestanding PTO films can be transferred to arbitrary substrates. d) RSM around the STO (103) peak and (013) peak for epitaxial PTO/SAO/STO films. e) XRD 2*θ*‐*ω* scan of epitaxial PTO/SAO/STO films (blue) and freestanding PTO films (purple). f) Results of optical SHG measurements of epitaxial PTO/SAO/STO (blue) and freestanding PTO (purple) films with *p*‐out configurations and *s*‐out configurations (dots are experimental data and lines are fitting curves). SHG fitting results reveal that the epitaxial PTO/SAO/STO films exhibit tetragonal phase with a 4 *mm* point group whereas the freestanding PTO films exhibit orthorhombic‐like (O‐like) phase with an *mm*2 point group.

The epitaxial PTO/SAO/STO films were prepared by depositing water‐soluble sacrificial SAO layers (≈60 nm) followed by ferroelectric PTO layers (≈50 nm) on (001)‐oriented single‐crystal STO substrates using the pulsed laser deposition (PLD) technique (**Figure** **1**a). Subsequently, the sacrificial SAO layers were completely dissolved in deionized water, releasing the PTO films from the STO substrates. These freestanding PTO films could then be transferred onto arbitrary substrates (Figure 1b,c). More details of the lift‐off process can be found in the Experimental section and the Supporting Information (Figure S1 and Table S1, Supporting Information).

Consistent with the general knowledge that the in‐plane lattice constants of the bulk PTO (3.902 – 3.904 Å) are similar to those of the STO (3.905 Å) substrates,^[^ ^40^, ^47^ ^]^ our results showed that the epitaxial PTO films were almost coherently grown on the SAO capped STO (001) substrates (Figure 1d), where the in‐plane lattice constant of SAO (≈3.904 Å, in pseudo‐cubic notation) was almost the same with that of STO.^[^ ^43^, ^48^ ^]^ The sharp PTO (00*l*) XRD peaks of epitaxial PTO/SAO/STO films revealed that the films exhibited the high‐quality of (001) single‐crystallinity (Figure 1e). Moreover, a large tetragonality *c*/*a≈*1.067 of PTO was observed in the epitaxial films (Table S1, Supporting Information), implying the large off‐centering and high polarizability. To further validate the crystal structure, SHG measurements were performed with the *p*‐out and the *s*‐out configurations where the analyzer polarization directions were parallel and perpendicular to the light incidence plane (Figure 1f), respectively. The SHG *p*‐out features exhibited a twofold rotational symmetry with two major peaks at 0° and 180°, as well as two minor peaks at 90° and 270°, while the SHG *s*‐out features exhibited a fourfold rotational symmetry with the same intensity of peaks at ≈45°, 135°, 225°, and 315°. These observations are typical characteristics of tetragonal crystal structures.^[^ ^49^, ^50^, ^51^ ^]^ By fitting the SHG features, we addressed that the epitaxial PTO films possessed a standard tetragonal phase with a 4 *mm* point group (Figure 1f). These findings collectively demonstrated the high quality of the epitaxial PTO/SAO/STO films.

On the other hand, no peaks for SAO but only sharp peaks for PTO (00*l*) were observed in XRD 2*θ*‐*ω* scan after dissolving the SAO layers, indicating that the freestanding PTO films maintained the high quality of single crystallinity (Figure 1e). However, after releasing the strain from the STO substrates, the out‐of‐plane lattice constants of freestanding PTO films decreased while the in‐plane ones increased (Table S1, Supporting Information). As a result, compared to the epitaxial PTO/SAO/STO films, the tetragonality, *c*/*a*, of the freestanding PTO films decreased (from 1.067 to 1.039), accompanying with a decrease of the SHG intensity (Figure 1f). As shown in Figure 1f and Figure S2 (Supporting Information), we presented both tetragonal and orthorhombic fitting curves for the *s*‐out data of the freestanding PTO films. Our fitting analysis revealed that the fitting of an orthorhombic phase with an *mm*2 point group exhibited a smaller residual standard deviation (σ≈568.3) and a larger coefficient of determination (R^2^≈0.964) compared to the fitting of a tetragonal phase with a 4 *mm* point group (σ≈654.5, R^2^≈0.952). We also observed that the 2*θ* of (101) peaks in freestanding PTO films was 0.1° smaller than that in epitaxial ones, indicating a possible phase transition from the tetragonal phase to the orthorhombic phase.^[^ ^52^ ^]^ In summary, based on our theoretical simulation of the SHG data, we deduced that the freestanding PTO films exhibited an orthorhombic‐like phase with an *mm*2 point group. This conclusion was consistent with previous calculation results that the metastable orthorhombic phase (space group Amm2 with the polar axis along the [110] direction) was stabilized under tensile strain.^[^ ^53^, ^54^ ^]^ In our work, the orthorhombic‐like phase is probably resulted from releasing a minor compressive lattice mismatch (≈−1.3%) between PTO layers and STO substrates,^[^ ^55^ ^]^ suggesting a partial transition of ferroelectric order orientation from out‐of‐plane to in‐plane when the PTO layers were freestanding.

Furthermore, using optical microscopy and atomic force microscopy (AFM), we characterized the topography of the freestanding PTO films transferred onto Pt‐coated Si wafers (Figure S12, Supporting Information). The freestanding PTO nanosheets exhibited wrinkled surfaces with 30 nm‐thick protrusions along the [100] and [010] directions (Figure S12d,f, Supporting Information), which might result from the increase of in‐plane lattice constants. The formation of wrinkles was caused by the relaxation of the local stress in freestanding films. These regular patterns of these wrinkled surfaces could be advantageous for the development of self‐assembled flexible electronics based on the flexoelectric effect.^[^ ^56^ ^]^

### 2.2 Ferroelectric Properties of PTO Films

Ferroelectric order of PTO films is undoubtedly affected by the structure evolution discussed above. It is well‐known that electric dipoles emerge in symmetry‐breaking structures, and the spontaneous ordering of the dipoles contributes to a macroscopic polarization, which is switchable under external electric fields.^[^ ^57^ ^]^ To investigate the variation of the ferroelectric order before and after the lift‐off process, electrodes were set on both the top and the bottom of the PTO layers. Specifically, the conducting probe was utilized as the top electrode, and a metallic SrRuO_3_ (SRO) layer, known for its compatibility with most perovskite oxides,^[^ ^58^ ^]^ was epitaxially grown as a bottom electrode. Consequently, the epitaxial PTO/SRO/SAO/STO films and the freestanding PTO/SRO bilayers were prepared with the thickness of the ferroelectric PTO layer to be 50 nm, the bottom electrode SRO layer to be 30 nm, and the sacrificial SAO layer to be 60 nm, respectively (see the Experimental section and more details in Figure S3 and Table S1, Supporting Information).

**Figure 2:**
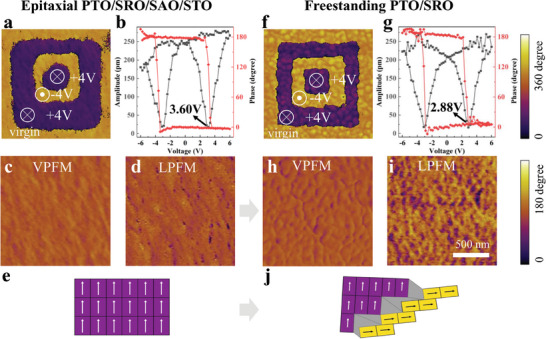
Ferroelectricity of epitaxial PTO/SRO/SAO/STO films and freestanding PTO/SRO bilayers. a,f) PFM phase images with drive voltages of +4 V, ‐4 V, and +4 V applied on square patterns. To be noted, the outermost rings were the virgin domains. b,g) PFM phase (red) and amplitude (black) curves as a function of voltage. c,h) Vertical‐PFM (VPFM) phase and d,i) lateral‐PFM (LPFM) phase images. e) Schematic images of out‐of‐plane polarization. j) Schematic images of the coexistence of out‐of‐plane and in‐plane polarization. Images of a, c, d, f, h, and i have the same scale bar of 500 nm. The thicknesses of PTO, SAO, and SRO layers are 50 nm, 60 nm, and 30 nm, respectively.

The ferroelectric switching behaviors and virgin domains of both the epitaxial and freestanding films were probed by PFM (**Figure** **2**; Figure S4, Supporting Information). With ±4 V voltages applied on a 1 µm × 1 µm area, both the epitaxial and freestanding bilayers exhibited robust ferroelectricity accompanied by a 180° phase reversal (Figure 2a,b,f,g). Meanwhile, roughly the same amplitude of ≈250 pm was measured in epitaxial films and freestanding bilayers, indicating well‐maintained piezoelectric properties after strain release (Figure 2b,g).

Ferroelectric domains, known as microscopic regions with aligned spontaneous polarization, are essential to ferroelectricity.^[^ ^59^ ^]^ We found that both the epitaxial and freestanding bilayers showed a spontaneous macroscale upward polarization in virgin domains (Figure 2a,f). Additionally, upon comparing the PFM phase image in the epitaxial films (Figure 2c,d) with those in the freestanding bilayers (Figure 2h,i), we observed an increased presence of dark regions in the freestanding bilayers, indicating the existence of in‐plane domains. It is worth noting that topography can introduce interference in the phase mapping of PFM, and the possibility of scan artefacts in rough regions cannot be ruled out (as illustrated by the white curves in Figure S4, Supporting Information). Nevertheless, significant phase differences were still observed in flat regions (roughness RMS <0.8 nm) of freestanding bilayers, whereas the phase in a similar region of epitaxial films remained almost unchanged (Figure S4, Supporting Information). Consequently, we concluded that both out‐of‐plane and in‐plane domains exist in freestanding PTO/SRO bilayers. In contrast, two types of epitaxial films (PTO/SRO/SAO/STO films and PTO/SRO/STO films) only exhibited out‐of‐plane domains (Figure S5, Supporting Information). This observation suggests that upon releasing the compressive strain from the substrates, the ferroelectric order transited from out‐of‐plane polarization to the in‐plane polarization in the freestanding PTO (50 nm) / SRO (30 nm) bilayers, possibly due to a decrease in the *c*/*a* ratio (from 1.080 to 1.054, Table S1, Supporting Information). The similar phenomenon was also observed in epitaxial PTO films under tensile strain, while the lattices of PTO were slightly orthorhombically distorted.^[^ ^55^, ^60^ ^]^

These different ferroelectric domain structures significantly impacted the electrical performance of PTO films including leakage current and driven switching voltage. The freestanding PTO/SRO bilayers exhibited sharp square borders in a 1 µm × 1 µm area with applying ±4 V voltages (Figure 2f), whereas the epitaxial PTO/SRO/SAO/STO films displayed rounded borders (Figure 2a), indicating lower leakage currents in the freestanding bilayers. Since the original written pattern was square with sharp edges, the rounded borders in the epitaxial PTO/SRO/SAO/STO films were probably resulted from the leakage currents, while the sharp square borders in the freestanding PTO/SRO bilayers exhibited a good preservation of written patterns. Moreover, lower switching voltage (≈2.88 V) were needed for freestanding bilayers than that (≈3.60 V) for the epitaxial ones (Figure 2b,g), suggesting the reduced energy consumption for switching the polarization of the freestanding bilayers. As the previous calculations showed that the energy barrier of the 180° switching (polarization turned from [001] to [00$\bar{1}$] direction by a tetragonal‐orthorhombic‐tetragonal phase transition) was much larger than that of the 90° switching (polarization turned from [110] to [001] direction by an orthorhombic‐tetragonal phase transition),^[^ ^61^ ^]^ we think the lower switching voltage in freestanding bilayers was probably resulted from the lower energy barrier in an orthorhombic‐tetragonal phase transition. Additionally, the strain effect arising from different layer thicknesses of PTO and SRO layers in epitaxial films were also discussed (Figures S6 and S7, Supporting Information). A decreased thickness of SRO layer (from 30 to 10 nm) resulted in more virgin in‐plane domains in freestanding PTO (50 nm) / SRO (10 nm) bilayers, as the SRO layer was too thin to limit the evolution of ferroelectric order from out‐of‐plane to in‐plane in PTO layer. Besides, these freestanding PTO/SRO bilayer films have a high breakdown electric field of 2.8 MV/cm (Figure S6, Supporting Information). On the other hand, with decreasing thickness of PTO layer from 50 to 30 nm, only out‐of‐plane domains but rarely in‐plane domains were observed in the freestanding PTO (30 nm) /SRO (30 nm) bilayers (Figure S7, Supporting Information). This behavior could be attributed to the limited lattice relaxation of residual strain and that out‐of‐plane domains rather than in‐plane domains preferred to form in the thinner PTO films.^[^ ^62^ ^]^ In short, the ferroelectric order transition from out‐of‐plane domains to in‐plane domains was closely affected by the thickness of the ferroelectric layer, which was observed in thick freestanding films (50 nm‐thick for PTO) but not in thin ones (30 nm thick for PTO). All in all, these freestanding PTO/SRO bilayers with robust ferroelectricity, low switching voltage, and high breakdown electric fields are beneficial to the potential applications in long‐lifetime nanodevices.

### 2.3 Large Tensile Strain Applied in Flexible Freestanding PTO Films

With the removal of substrate constraints, property manipulation is allowed for freestanding films by applying large and continuously tunable strain, which is a significant advantage compared to the epitaxial films grown on substrates. It has been reported that the out‐of‐plane lattice constants decreased but the in‐plane lattice constants increased after applying uniaxial tensile strain in freestanding PTO films, giving rise to weakened ferroelectricity.^[^ ^40^ ^]^ However, it remains a challenge to clarify the dynamic ferroelectric evolution of freestanding PTO films in response to strain when considering both out‐of‐plane and in‐plane domains. Here, using the optical SHG measurement, we revealed the physical relationships among strain, crystal structure, and dynamic ferroelectric order in freestanding PTO films.

**Figure 3:**
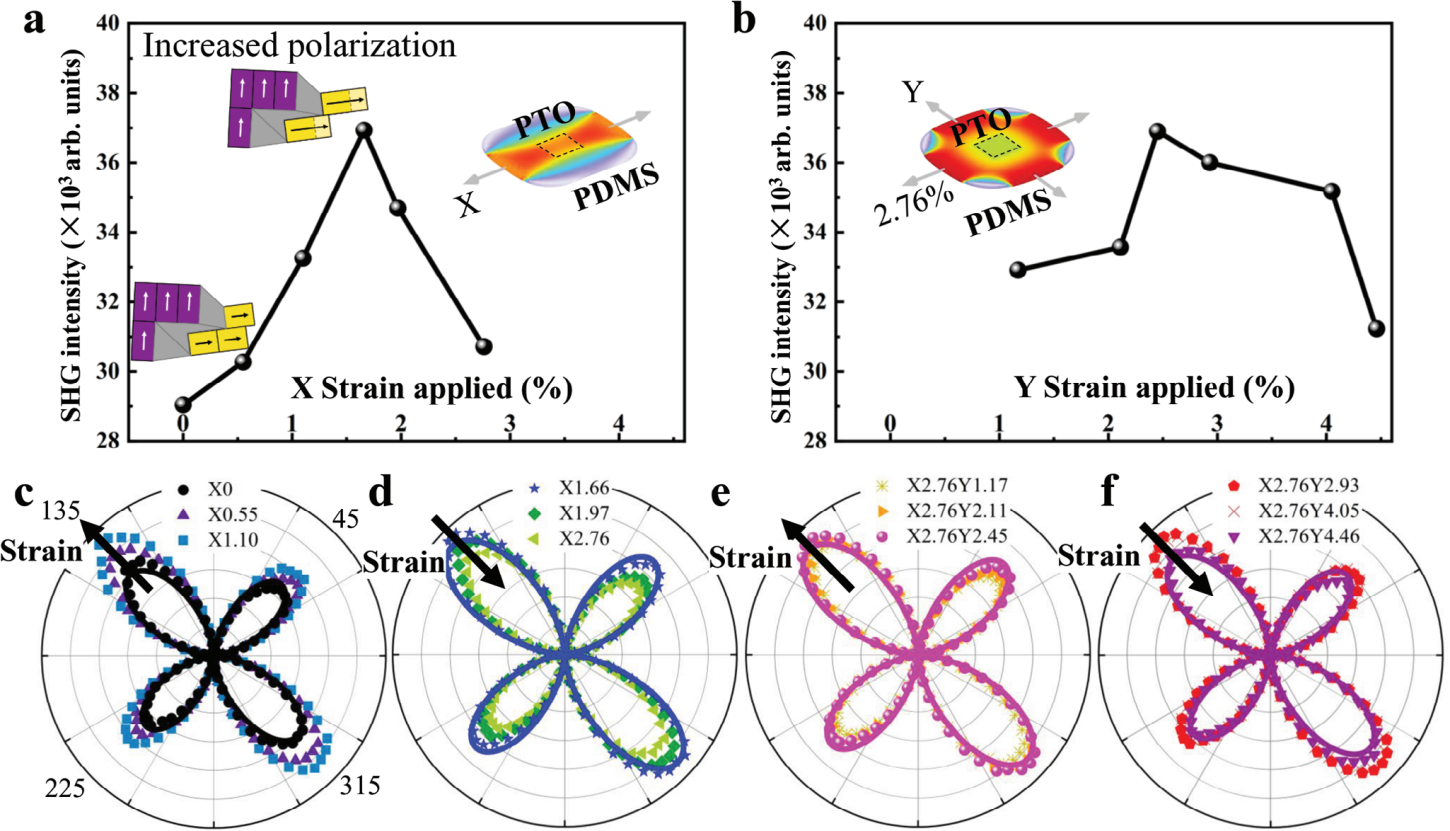
SHG evolution for 50 nm‐thick freestanding PTO films with increasing uniaxial and biaxial tensile strain applied on polymer PDMS substrates. a,b) Maximum SHG intensity (measured with *p*‐out configuration) of freestanding PTO films (transferred onto the flexible polymer PDMS substrate) as a function of the uniaxial tensile strain (up to 2.76% along *x*‐axis direction) and of the biaxial tensile strain (2.76% along *x*‐axis direction and up to 4.46% along *y*‐axis direction, respectively). c‐f) SHG anisotropy polar plots measured with *s*‐out configuration for freestanding PTO films.

The maximum SHG intensity was extracted from the results with the *p*‐out configuration and was used to analyze the ferroelectric polarization evolution of the freestanding PTO films (**Figure** **3**a,b; see the Experimental section and more details in Figure S8, Supporting Information), and the anisotropy of SHG results with the *s*‐out configuration was analyzed to reveal the evolution of structures (Figure 3c–f). First of all, the 50 nm‐thick freestanding PTO films on flexible polymer polydimethylsiloxane (PDMS) substrates were stretched along the *x* axis ([100] direction) with increasing uniaxial tensile strain of 0, 0.55%, 1.10%, 1.66%, 1.97%, and 2.76% (labeled as X2.76 for example) in turn (Figure 3a,c,d). We observed that the SHG intensity initially increased with the increased tensile strain up to 1.66%. It is worth noting that the reported tensile strain values in our study specifically refer to those applied directly to the flexible polymer substrates. The tensile strain calculated as (*l*‐*l*_0_)/*l*_0_, where *l*_0_ and *l* referred to the side lengths of unstretched and stretched freestanding PTO films, respectively. These strain values may not be fully transferred to the attached PTO layers. Consequently, we conducted a supplementary experiment on X‐ray diffraction intensity as a function of tensile strain (Figure S9, Supporting Information). The results revealed a slight reduction in the lattice constant *c* with increasing tensile strain. Generally, a decrease in the out‐of‐plane lattice constant *c* usually indicates an increase of the in‐plane lattice constant *a*.^[^ ^40^ ^]^ This variation of the lattice constants likely contributed in the increased space symmetry breaking in the freestanding PTO, subsequently leading to the enhancement of SHG intensity. However, as the strain further increased from 1.66% to 2.76%, the SHG intensity decreased. The reasons for the decreased SHG intensity might be attributed to the possible formation of microcracks, overstressing against the surface adhesion, or potential destruction to the domain structures due to excessive applied strain. Then, maintaining 2.76% tensile strain along the *x* axis, tensile strain of 1.17%, 2.11%, 2.45%, 2.93%, 4.05%, and 4.46% (labeled as X2.76Y4.46 for example) were applied along the *y* axis ([010] direction) in turn (Figure 3b,e–f). The SHG intensity increased again with small tensile strain along the *y* axis up to 2.45% and then decreased with the increased strain along the *y* axis up to 4.46%, similar to the stretching along the *x* axis. Fortunately, even under the large tensile strain along the *y* axis of 4.46%, the SHG intensity was still a little bit higher than that in the non‐stretched state, showing the well‐preserved ferroelectricity. The phenomenon that the polarization increased first and then decreased with increasing strain was also observed in SHG spectra and SHG measurements with the *p*‐out configuration (Figure S8, Supporting Information).

Additionally, whether under uniaxial or biaxial stretching conditions, the SHG *s*‐out features always exhibited fourfold rotational symmetry and the intensities of peaks at ≈135° and 315° were larger than those at ≈45° and 225°, indicating that the freestanding PTO films maintained orthorhombic‐like phase (Figure 3c–f). By fitting SHG results, the orthorhombic‐like phase was confirmed with an *mm*2 point group at the initial non‐stretched state (X0), the two states with maximum SHG intensities (X1.66 and X2.76Y2.45), and the final strain state (X2.76Y4.46) (Figure 3c–f).

### 2.4 Ferroelectric Order Evolution of PTO Films with Temperature

**Figure 4:**
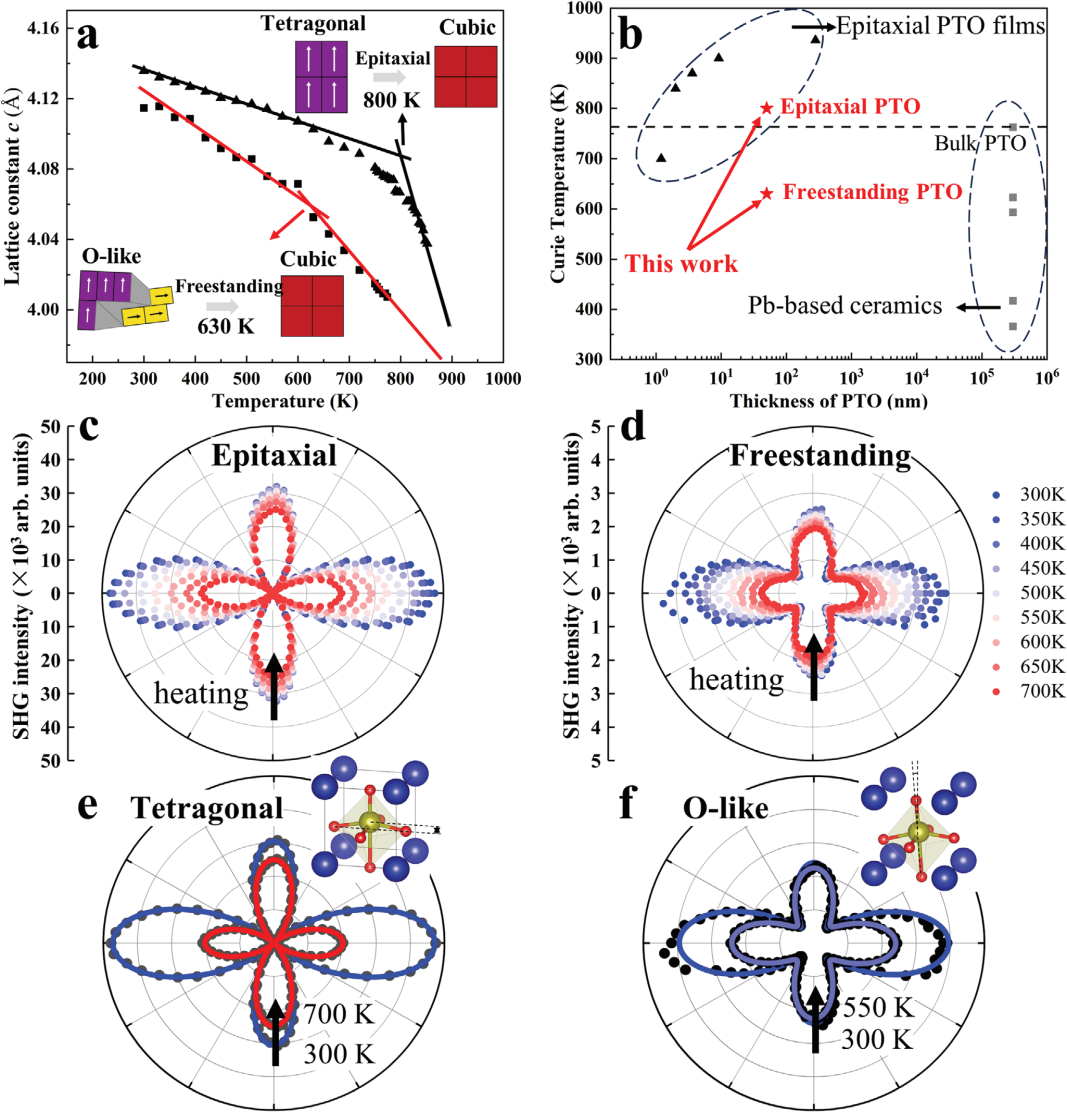
Structural and ferroelectric evolution with varying temperature of epitaxial PTO/STO films and freestanding PTO films. a) Out‐of‐plane lattice constants of the epitaxial films and those of the freestanding films above 300 K. The inset diagrams illustrate the phase transitions from ferroelectric phases to paraelectric phases. b) Curie temperature (*Tc*) of PTO comparing with previous works of Pb‐based ferroelectrics.^[^ ^66^, ^67^, ^68^, ^69^ ^]^ c,d) SHG anisotropy polar plots measured with *p*‐out configuration from 300 K to 700 K in the epitaxial PTO/STO films and in the freestanding PTO films. e,f) By analyzing SHG patterns below the *Tc*, a tetragonal phase with a 4 *mm* point group is confirmed in the epitaxial PTO/STO films, whereas an orthorhombic‐like phase with an *mm*2 point group is confirmed in the freestanding PTO films.

On the other hand, high temperature is a key challenge in the development of nano‐scale and flexible electronics, especially in the fields of deep earth drilling, avionics, and space exploration.^[^ ^63^, ^64^ ^]^ To explore the evolution of the structure and ferroelectric order with varying temperature, in this work, XRD 2*θ*‐*ω* scans and SHG measurements were both performed on 50 nm‐thick epitaxial PTO/STO films and freestanding PTO films with varying temperature (300 K‐880 K, **Figure** **4** and see details in Figures S10 and S11, Supporting Information).

Our results showed that the (002) XRD peak of PTO shifted to higher 2*θ* angles with increasing temperature, indicating a decrease of the out‐of‐plane lattice constants according to Bragg's law for both epitaxial PTO/STO films (from 4.136 to 4.010 Å) and freestanding PTO films (from 4.119 to 3.971 Å) (Figure 4a). This can be attributed to the negative thermal expansion effect in PTO.^[^ ^65^ ^]^ Moreover, it was observed that the SHG intensity decreased with increasing temperature, suggesting a decrease of polarization (Figure S11, Supporting Information).

There were several differences between the results of the freestanding PTO films and those of the epitaxial PTO/STO ones. First, the out‐of‐plane lattice constants of the freestanding PTO films were consistently lower than those of the epitaxial films throughout the temperature range (Figure 4a), which indicated that the out‐of‐plane polarization of the PTO became slightly weaker after the lift‐off process, resulting in the lower SHG intensity of the freestanding PTO films. The out‐of‐plane lattice constants of the freestanding PTO films exhibited a sharp drop, while those of the epitaxial films showed a gradual drop. This difference suggested that the presence of STO substrates before releasing limited the negative thermal expansion behavior of the PTO layers. In other words, the lattice constants of cubic STO substrates increased (from 3.917 to 3.935 Å) with increasing temperature, while those of PTO decrease with increasing temperature (Figure S10, Supporting Information), so that the competition between them weakened the thermal expansion effect.

The *Tc* of the freestanding PTO films was ≈630 K, obtained from SHG measurement (Figure S11, Supporting Information) and confirmed by XRD analysis (Figure 4a). This *Tc* was comparable to that of many ferroelectric ceramics such as Pb(Zr_,_ Ti)O_3_ ceramics^[^ ^66^, ^67^ ^]^ (Figure 4b). By analyzing the SHG results of freestanding PTO films, we found that these films maintained an orthorhombic‐like phase with an *mm*2 point group below 630 K (Figure 4d,f) and transformed into a cubic phase with zero SHG intensity above 630 K (Figure S11, Supporting Information). Meanwhile, the *Tc* of the epitaxial PTO/STO films was ≈800 K, deduced from the XRD measurement (Figure 4a), which was a little bit higher than that of bulk PTO^[^ ^47^ ^]^ and fell in the range that the previous works have reported in the epitaxial PTO films^[^ ^68^, ^69^ ^]^ (Figure 4b). It is also found that the SHG intensity of the epitaxial films decreased linearly with the increasing temperature from 300 to 773 K, although the exact *Tc* of the epitaxial films were not detected by SHG due to the limitations of temperature range (Figure S11, Supporting Information) of our SHG system. By fitting the SHG results of the epitaxial PTO/STO films, a tetragonal phase with a 4 *mm* point group was maintained below 800 K (Figure 4c,e). Overall, the high *Tc* of ≈630 K in the freestanding PTO films with a thickness of only 50 nm suggests potential applications in wide‐temperature‐range and flexible electronic devices in the future.

## 3 Conclusion

In conclusion, we systematically investigated the evolution of ferroelectric order under continuous variation of the tensile strain and temperature for both freestanding PTO films and epitaxial ones. Our results revealed that there are only out‐of‐plane domains in the tetragonal epitaxial PTO films. By contrast, both out‐of‐plane and in‐plane domains were observed in the orthorhombic‐like freestanding PTO films. In addition, the optical SHG measurements were applied to explore the effect of continuously varying tensile strain on the ferroelectric order of freestanding PTO films. We observed strengthened polarization of the freestanding films within small tensile strain from 0% up to 1.66%. Remarkably, even under large biaxial in‐plane tensile strain up to 2.76% along the *x*‐axis and up to 4.46% along the *y*‐axis, the freestanding PTO films maintained their orthorhombic‐like phases and well‐preserved ferroelectricity, suggesting their potential applications for flexible devices. Furthermore, our observation of SHG and XRD signals as a function of temperature extracted a high *Tc* (≈630 K) in the freestanding PTO films with the thickness of only 50 nm, indicating the suitability of these films for wide‐temperature‐range electronic nanodevices. Our study not only contributes to the fundamental understanding of the structure and the dynamic ferroelectric order of freestanding PTO films but also highlights their potential applications for the next‐generation electronic nanodevices with flexibility and thermostability.

## 4 Experimental Section

### Preparations of Epitaxial and Freestanding films

Epitaxial PTO/STO, SAO/STO, and SRO/STO films were coherently grown on (001)‐oriented STO substrates, respectively, by the PLD system with XeCl excimer laser (λ = 308 nm). Laue oscillations were clearly visible in the XRD of these films (Figure S1, Supporting Information), suggesting their high crystallinity and sharp interfaces. Specifically, PTO layers were deposited under conditions of oxygen partial pressure of 20 Pa, deposition temperature of 600 °C, laser energy of 1.2 J cm^−2^, and repetition rate of 4 Hz, respectively, whereas the corresponding conditions for SAO layers were 5 Pa, 780 °C, 1.2 J cm^−2^, and 7 Hz, respectively, as well as those for SRO layers were 10 Pa, 650 °C, 1.2 J cm^−2^, and 4 Hz, respectively. In this work, the growth conditions for the PTO, SAO, and SRO layers in all kinds of films were the same as those mentioned above. For example, the epitaxial PTO/SAO/STO films were fabricated with the SAO layer first deposited followed by the PTO layer on the top. Besides, the epitaxial PTO/SRO/SAO/STO films with bottom electrode SRO were also fabricated on STO substrates using PLD system to characterize their ferroelectricity with PFM (Figure S3, Supporting Information). The growth rate of each layer was calculated and controlled precisely by counting the number of laser pulses. Unless otherwise stated, the thicknesses of the PTO layers, the SAO layers, and the SRO layers were 50, 60, and 30 nm, respectively.

The lift‐off process was followed after the epitaxial growth to prepare freestanding films. Taking epitaxial PTO/SAO/STO films as an example, the PTO layers were released from the STO substrates by selectively dissolving the sacrificial SAO layer with deionized water. The strain‐released freestanding PTO layers were reattached to the STO substrates through van der Waals attraction in the XRD and RSM measurements, minimizing additional stress. These freestanding PTO layers could also be transferred onto other substrates, such as silicon wafers or flexible polymers. For instance, the epitaxial PTO/SAO/STO films were first capped with a sheet of flexible PDMS polymer, pressed tightly, and immersed in deionized water. When the SAO layers were selectively dissolved, the STO substrates could be lifted off, leaving the freestanding PTO films on the flexible substrates. Continuous tensile strain was then applied to these freestanding PTO films. Here, the polymer substrates were fixed with screws and were stretched by a spiral micrometer with an accuracy of 0.01 mm. The tensile strain of the freestanding PTO films was calculated as (*l*‐*l*_0_)/*l*_0_, where *l*_0_ and *l* referred to the side lengths of unstretched and stretched freestanding PTO films, respectively.

### Structure Characterizations

Structure crystallography was analyzed at room temperature using XRD, RSM, and X‐ray reflectivity (XRR) measurements on the equipment of a Panalytical X'Pert3 MRD diffractometer with Cu Kα_1_ radiation (*λ* = 1.5406 Å) equipped with a 3D pixel detector. Here, XRD 2*θ*‐*ω* scan and RSM measurements were performed to determine both the out‐of‐plane and in‐plane lattice constants before and after the lift‐off process. The thicknesses of different films were obtained by fitting XRR curves using GenX software and were confirmed by AFM (MFP‐3D Asylum Research microscope) measurements. AFM was also be used to scan the surface morphology of PTO‐based films.

Structural crystallographic analysis at elevated temperature between 300 and 880 K was characterized by XRD (Rigaku Smartlab) using a Cu‐Kα target (*λ* = 1.5406 Å) equipped with a high‐temperature chamber providing variable temperature from room temperature to 1473 K. These results of PTO films at elevated temperature had deducted the influence of STO substrates and were fitted with Lorentz peak function.

### Ferroelectric Characterizations

Ferroelectric properties of PTO films were characterized using PFM measurements (MFP‐3D Asylum Research microscope) at room temperature. A Pt/Ti‐coated tip with a 2 N m^−1^ spring constant (Olympus AC240TM) was used in the PFM tests. Hysteresis loops were collected in the dual AC resonance tracking (DART) mode. During the domain writing, a voltage range of 2–14 V was applied on the tip. The virgin domains or 180° domains were recorded in the DART mode when a driving voltage 1 V AC was applied to the tip.

### Optical SHG Measurements

The incident laser beam was generated by the Maitai SP Ti:Sapphire oscillator produced by Spectra Physics, which produced a femtosecond pulsed laser with the incident light power kept at 50 mW and a center wavelength of 800 nm (pulse width 120 fs, frequency 82 MHz). Both the incident angle and the reflection angle were fixed at 45°, and the polarization direction of the incident light field was adjusted by the rotation of the half waveplate. The polarization configuration of the reflection light was fixed as *p* or *s* polarization, and the anisotropy patterns under different reflection polarization configurations were obtained by rotating the incident light polarization angle.

The optical second‐order susceptibility tensors of the tetragonal phase with a 4 *mm* point group and the orthorhombic phase with an *mm*2 point group for fitting rotational anisotropy SHG results (the mirror plane is aligned parallel to the incident plane) were shown in the previous report.^[^ ^50^ ^]^

The temperature‐variable stage used in the SHG measurements process was a heating stage with a long temperature‐range of 77–873 K. The change in SHG intensity was measured from 300 to 700 K with the polarization configurations of the incident and reflection light fixed both *p* and *s* polarization. The wide‐temperature‐range SHG anisotropy polar plots were measured every 50 K from 300 to 700 K.

### Statistical Analysis

The fitting results of anisotropy SHG were evaluated using the residual standard deviation (σ) and the coefficient of determination (R^2^) in multiple regression analysis. The σ and R^2^ represent the dispersion and goodness of fit of the model, respectively. Here are the formulas for calculating them.

(1) $$\sigma \;=\sqrt{\frac{\sum_{i=1}^{n}{\left(y_{i}-{\hat{y}}_{i}\right)}^{2}}{n}}$$

(2) $$R^{2}=\;1-\frac{\sum_{i\;=\;1}^{n}{\left(y_{i}-{\hat{y}}_{i}\right)}^{2}}{\sum_{i\;=\;1}^{n}{\left(y_{i}-\bar{y}\right)}^{2}}$$

where *y_i_* and ${\hat{y}}_{i}$ represent the observed values and fitted values, respectively. $\bar{y}$ represents the mean of observed values. *n* is the sample size. When testing the SHG anisotropy, data points were collected every 4° over a total rotation of 360°, resulting in a sample size of 91. Furtherly, as shown in Figure S2c,d (Supporting Information) the residuals were displayed using a normal distribution *N*(0, *σ* ^2^) with a mean value of 0 and variance σ^2^.

## Conflict of Interest

The authors declare no conflict of interest.

## Author Contributions

S.H. and S.X. contributed equally to this work. These samples were grown and processed by S.S.H. under the guidance of K.J.J.; all S.H.G. measurements were conducted by S.X. under the guidance of K.J.J.; X.R.D. and P.F.M. experiments were performed by S.S.H. under the guidance of K.J.J.; S.X. and C.M. worked on the structural analysis and provided schematic pictures of a unit cell. P.Z.L., E.J.G., C.G., C.W., X.L.X., M. H., and G.Z.Y. participated in the discussions and provided important suggestions during the manuscript revision. K.J.J. initiated the research and supervised the work. K.J.J., S.S.H., S.X., and C.M. wrote the manuscript with inputs from all authors.

## Supporting information

Supporting Information

## Data Availability

The data that support the findings of this study are available from the corresponding author upon reasonable request.
