# Supplementary material for: Ferroelectric Order Evolution in Freestanding PbTiO3 Films Monitored by Optical Second Harmonic Generation
Source: Adv Sci (Weinh). 2024 Jun 24;11(32):2307571. doi: 10.1002/advs.202307571 (PMC11348163; doi:10.1002/advs.202307571)
Supplement: Supplementary file 1 — Supporting Information [file ADVS-11-2307571-s001.docx]

Ferroelectric Orders Evolution in Freestanding PbTiO_3_ Films Monitored by Optical Second Harmonic Generation

Sisi Huang^1,2,†^, Shuai Xu^1,2,†^, Cheng Ma^1,2^, Pengzhan Li^1^, Er-Jia Guo^1,2^, Chen Ge^1,2^, Can Wang^1,2,3^, Xiulai Xu^1,4^, Meng He^1^, Guozhen Yang^1,2^, Kuijuan Jin^1,2,3,*^

^1^ Beijing National Laboratory for Condensed Matter Physics, Institute of Physics, Chinese Academy of Sciences, Beijing 100190, China

^2^ University of Chinese Academy of Sciences, Beijing 100049, China

^3^ Songshan Lake Materials Laboratory, Dongguan, Guangdong 523808, China

^4^State Key Laboratory for Mesoscopic Physics and Frontiers Science Center for Nano-optoelectronics, School of Physics, Peking University, Beijing 100871, China

^†^ These authors contributed equally: Sisi Huang, Shuai Xu

^*^Author to whom correspondence should be addressed: [kjjin@iphy.ac.cn](mailto:kjjin@iphy.ac.cn)

CONTENTS

[1. Fabrications and Structural Analysis 2](#_Toc144046164)

[2. Ferroelectric Properties of PTO Films at Room Temperature 5](#_Toc144046165)

[3. Large tensile strain Applied in Flexible Freestanding PTO Films 9](#_Toc144046166)

[4. Structural and Ferroelectric Evolution of PTO Films at High Temperature 10](#_Toc144046167)

[5. Freestanding PTO films Transferred on Si wafers 12](#_Toc144046168)

**1.** **Fabrications and Structural Analysis**

At the beginning, epitaxial films including 50 nm-thick ferroelectric PTO/STO films, 60 nm-thick water-soluble sacrificed SAO/STO films, and 30 nm-thick bottom electron SRO/STO films were grown to make sure all of them could be able to coherently deposit on the STO substrates (**Figure S1**). Herein, the out-of-plane lattice constant *c* of PTO, calculated according to Bragg’s law, was ~ 4.139 Å, in the range of 4.030 - 4.154 Å reported in other works^[1, 2]^. Rather than the cubic one by other groups^[3]^, SAO forms a tetragonal phase with a large out-of-plane lattice constant *c* = 4.286 Å and its in-plane lattice constant *a* (~ 3.904 Å) was almost the same to that in STO substrates^[4, 5]^. The lattice constant *c* of SRO was about 3.945 Å, coherently growing in STO as reported^[6]^ (Figure S1).


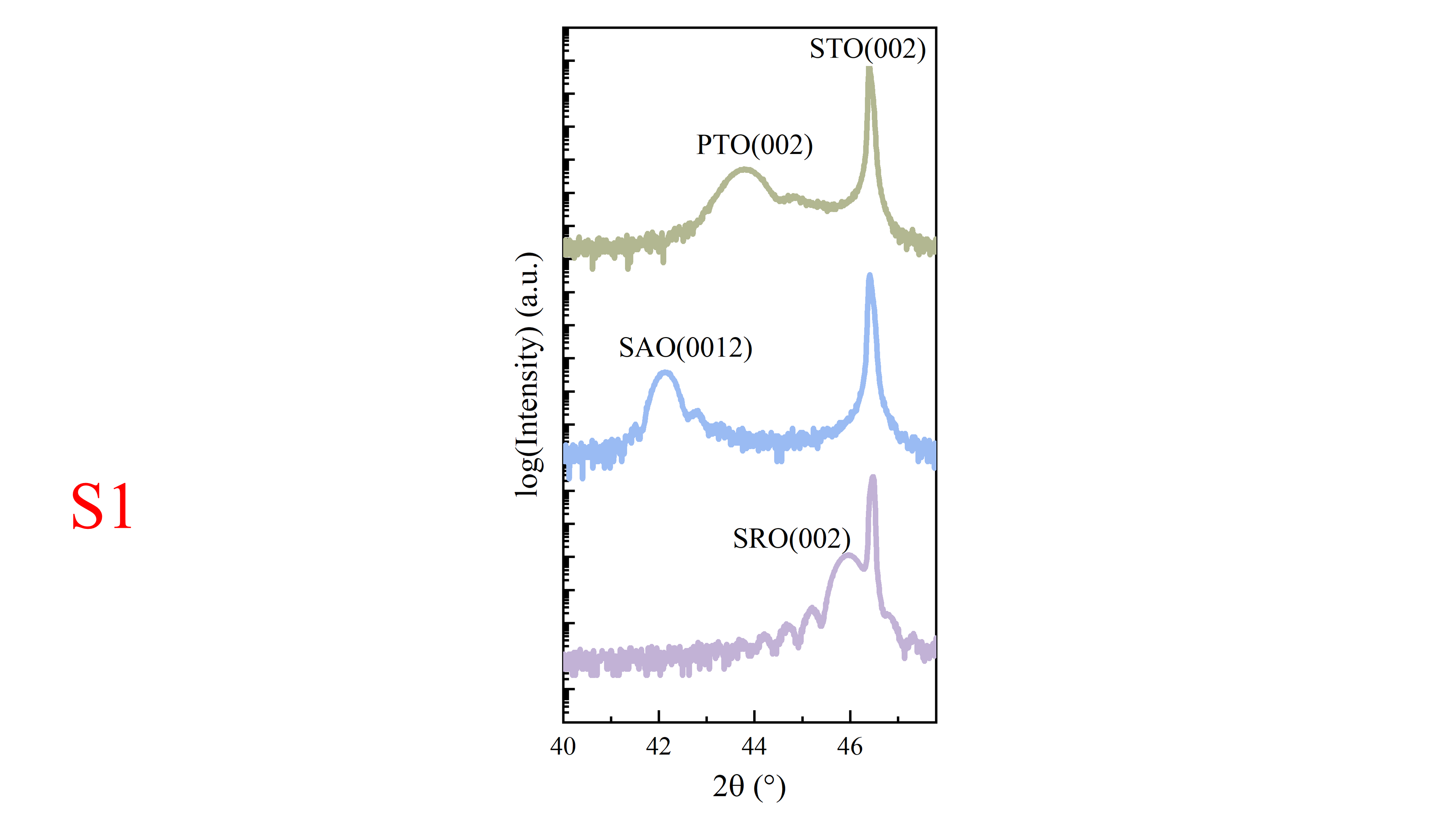


**Figure S1. XRD of epitaxial films.** PTO/STO films (top), SAO/STO films (middle), and SRO/STO films (bottom).


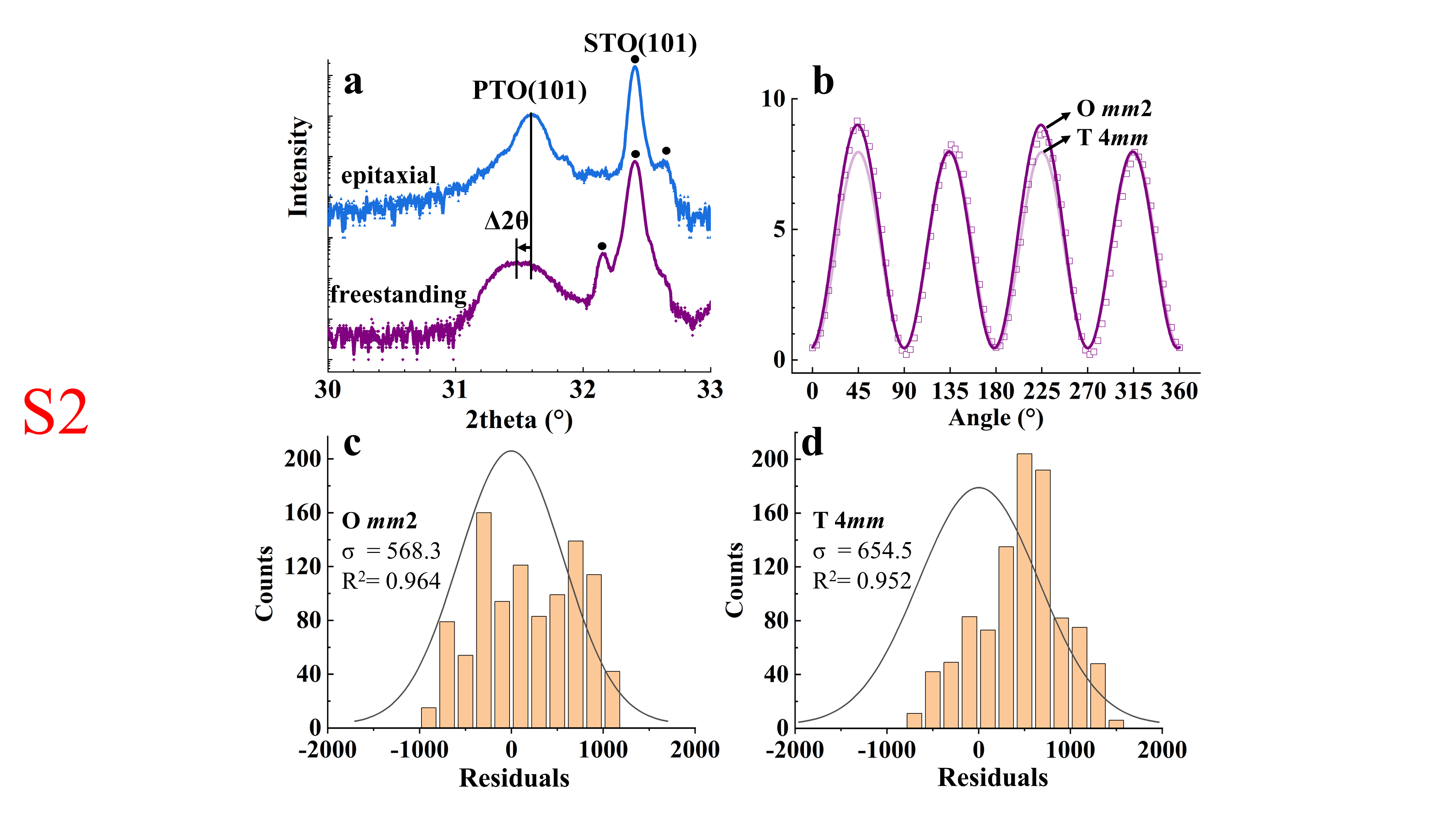


**Figure S2. XRD and SHG analysis for confirming the orthorhombic phase in freestanding PTO films.** a) XRD (101) peaks of epitaxial PTO/STO films (blue) and freestanding PTO films (purple), where the black dots mark the (101) peaks of the STO substrate. b) SHG *s*-out fitting results of freestanding PTO films with tetragonal fitting (T 4*mm*) and orthorhombic fitting (O *mm*2). c-d) The residual standard deviation (σ) and the coefficient of determination (R^2^) of SHG fitting results.

To explore ferroelectric order changes, the epitaxial PTO/SRO/SAO/STO films and freestanding PTO/SRO bilayers were prepared (**Figure S3** and **Table S1**). After dissolving the sacrificial SAO layers and releasing the strain, it was observed that a right-move of 2*θ* in PTO (002) peaks and up-move of ΔQz in both PTO (103) and (013) crystal indices, meaning the out-of-plane lattice constant *c* of PTO considerably decreased but the in-plane lattice constant *a* (or *b*) increased, which resulted in an increased volume of the unit cell (Table S1). This trend was the same with that in freestanding PTO films when releasing the strain from PTO/SAO/STO films (Figure 1). Besides, the full width at half maximum (FWHM) increased from 0.146° to 0.358°, which probably arises from the wrinkles induced by strain relaxation and macroscopic distortions during films transfer^[7, 8]^.


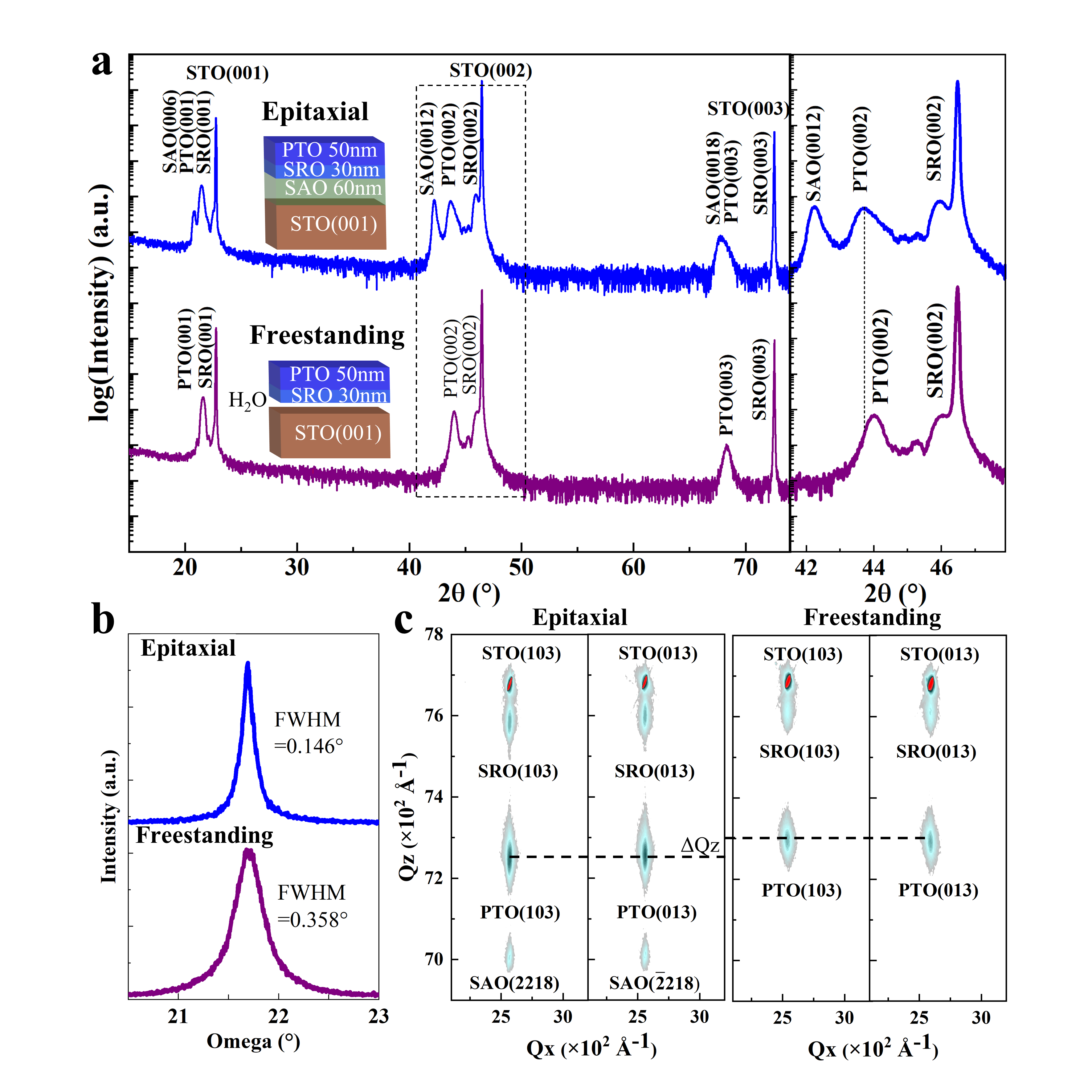


**Figure S3.** **Structural comparison between epitaxial PTO/SRO/SAO/STO films and freestanding PTO/SRO bilayers.** a) XRD results, the inset is schematic images of different layers in films. b) Rocking curves and c) RSM around (103) and (013) peaks of the epitaxial PTO/SRO/SAO/STO films (left) and the freestanding PTO/SRO bilayers (right).

**Table S1.** Crystal lattice constants of epitaxial PTO/SAO/STO films, freestanding PTO films, epitaxial PTO/SRO/SAO/STO films, and freestanding PTO/SRO bilayers, including lattice constants (*a*, *b*, and *c* of the PTO layers), volumes of the unit cells (*a*×*b*×*c*), and tetragonality (*c*/*a* and *c*/*b*). Here, the out-of-plane lattice constant (*c*) is obtained from (00*l*) peaks in XRD and the in-plane lattice constants (*a* and *b*) are obtained from (103) and (013) peaks in RSM.


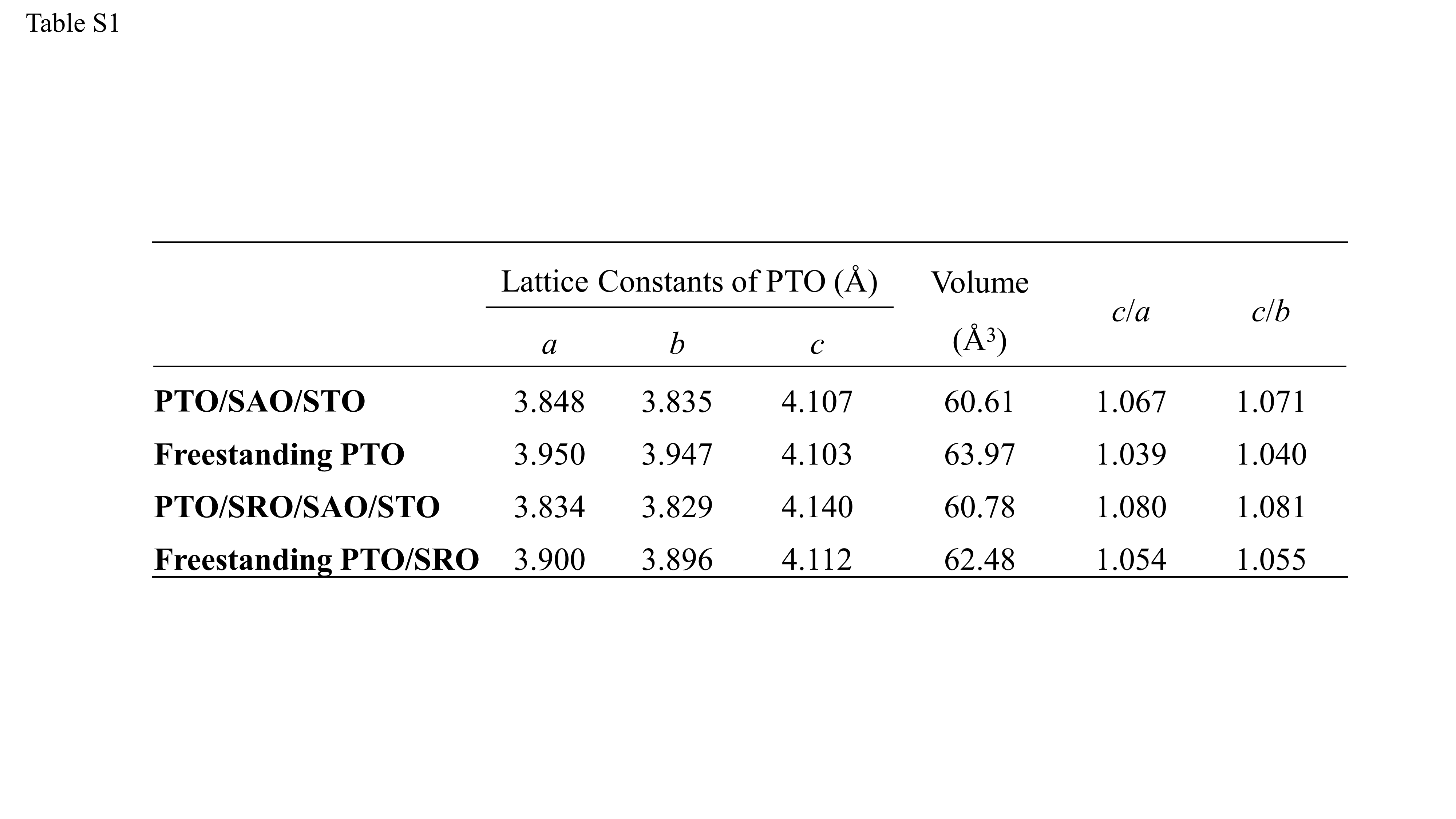


**2.** Ferroelectric Properties of PTO Films at Room Temperature

The epitaxial PTO/SRO/SAO/STO films, freestanding PTO/SRO bilayers (**Figure S4**), and epitaxial PTO/SRO/STO films (**Figure S5**) were prepared to conduct the PFM measurements. All of them have distinct ferroelectric 180° domains in out-of-plane direction under electric voltage ±4 V. Before applying electric voltage, a completely out-of-plane domains with out-of-plane polarizations were observed in virgin domains of epitaxial PTO/SRO/STO films (Figure S5), whereas dominated out-of-plane domains were observed in PTO/SRO/SAO/STO films. Furthermore, an increased proportion of in-plane domains were observed in freestanding PTO/SRO bilayers. As shown in Figure S4, we identified the boundaries of undulating steps in freestanding films with white curves, and analyzed the height and LPFM-phase at the flat regions at the middle of a step, denoted as A1 and A2. These regions had a height difference less than 0.8 nm. We observed a significant phase difference in the A2 region of freestanding films, whereas the phase in the A1 region of epitaxial films remained nearly unchanged. This observation suggests the coexistence of out-of-plane and in-plane domains in localized regions of the freestanding films. It is probably due to its increased in-plane lattice constants after releasing the strain from STO substrates.


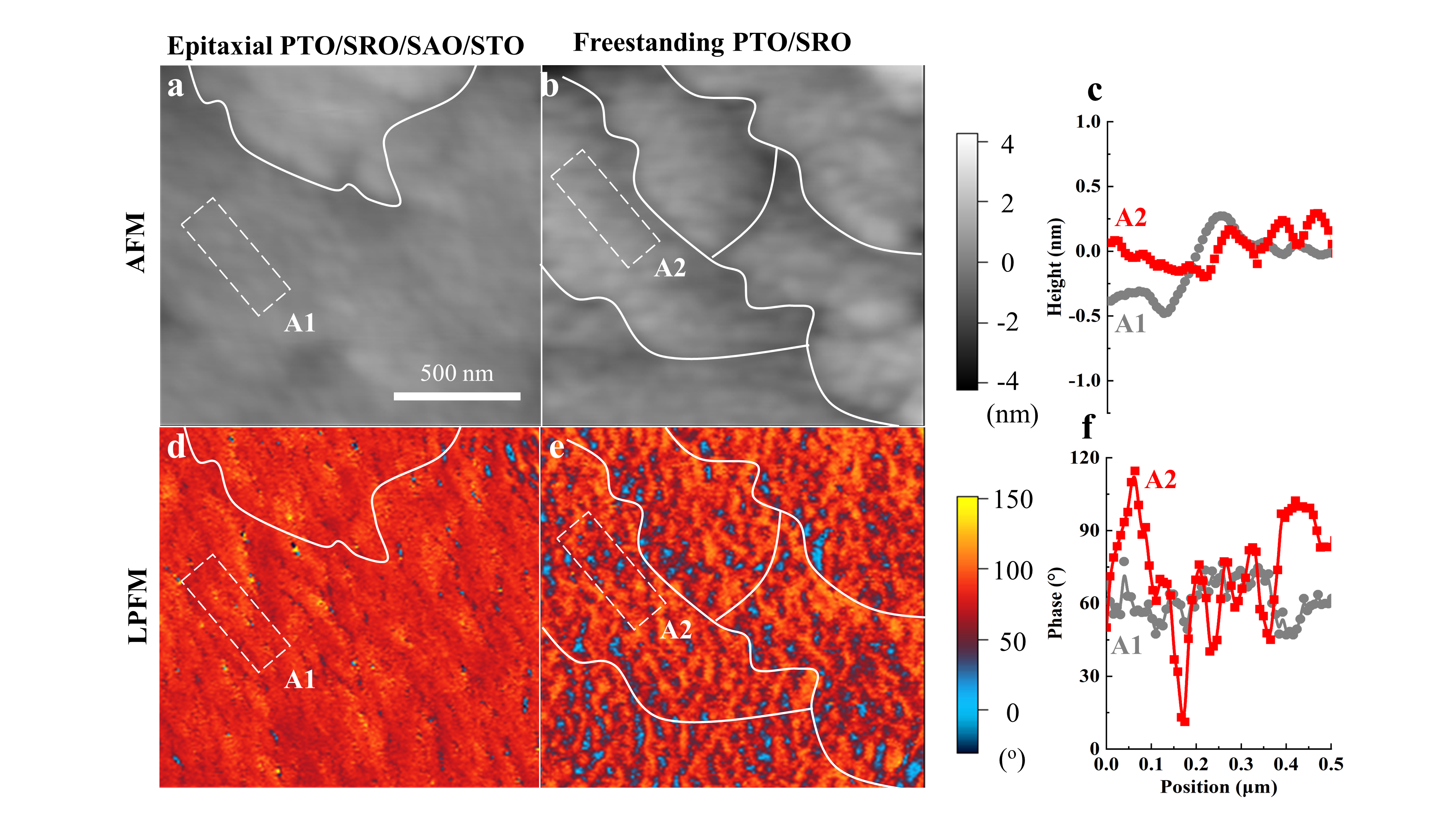


**Figure S4.** **AFM and LPFM of epitaxial PTO/SRO/SAO/STO films and freestanding PTO/SRO bilayers.** a-c) Topography from AFM. d-f) LPFM-phase images. Here, A1 and A2 donate flat regions at the middle of a step. The boundaries of these steps marked by white curves.


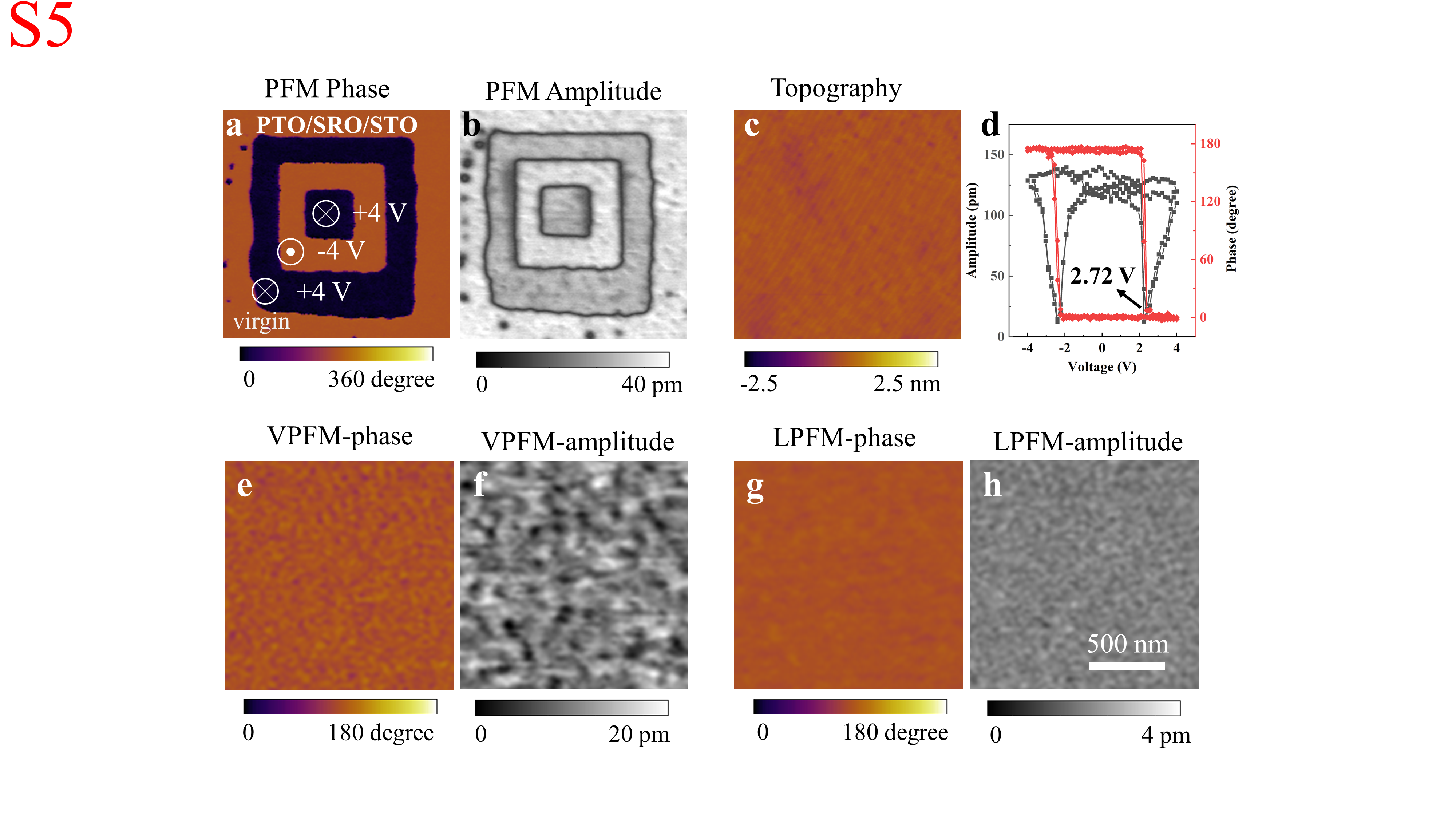


**Figure S5.** **Ferroelectricity of the epitaxial PTO/SRO/STO films.** a,b) Out-of-plane PFM phase and amplitude. c) Topography from AFM. d) hysteresis loops. e,f) Vertical-PFM phase and amplitude. g,h) Lateral-PFM phase and amplitude. The thicknesses of PTO and SRO layers are 50 nm and 30 nm respectively. Scale bar is 500 nm.


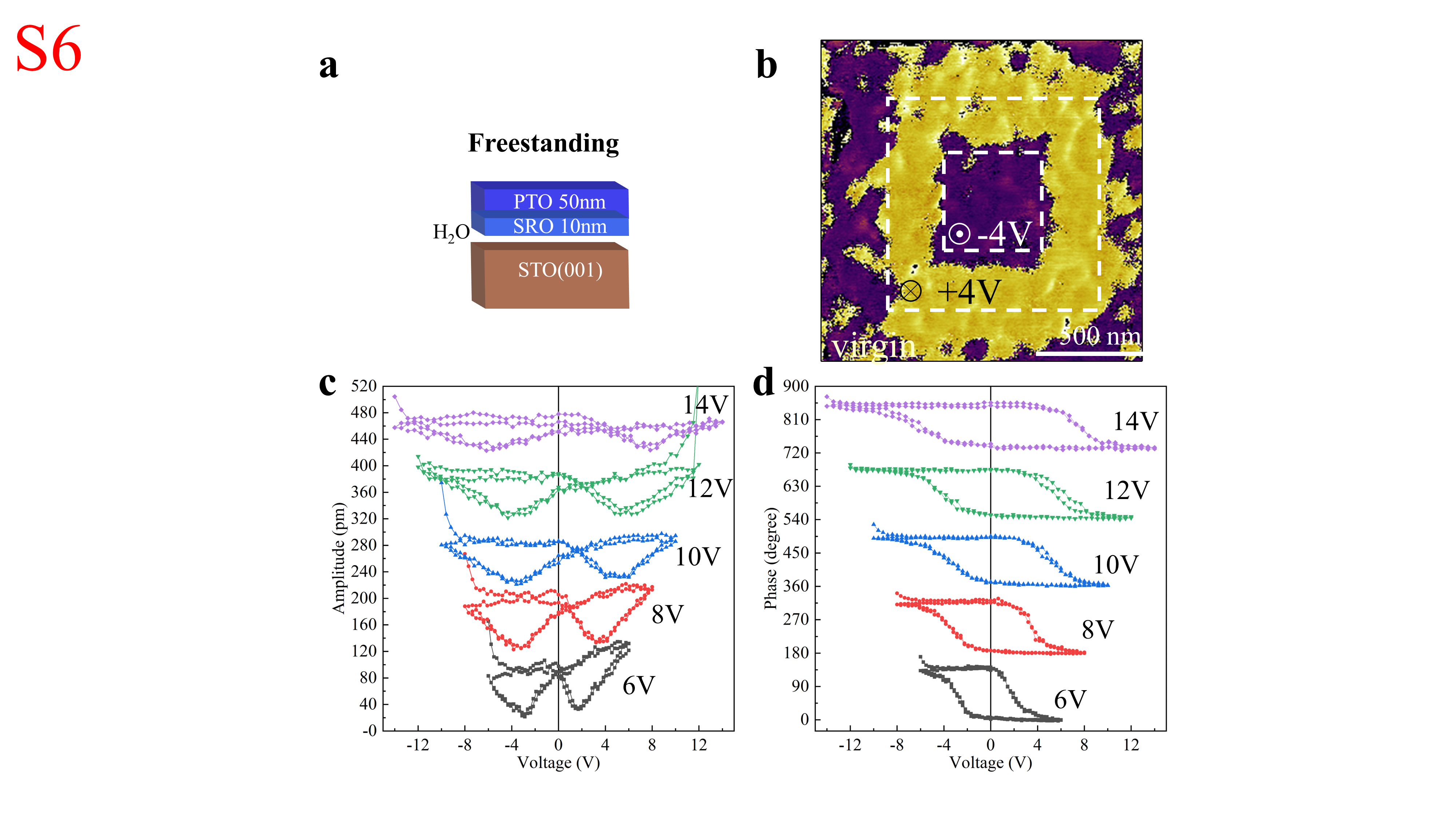


**Figure S6. Ferroelectricity of freestanding PTO (50 nm) / SRO (10 nm) bilayers**. a) Schematic structures of freestanding PTO/SRO bilayers. b) Out-of-plane PFM phase indicated its ferroelectricity with scale bar is 500 nm. c-d) Amplitude-voltage and phase-voltage hysteresis loops show the freestanding bilayers can withstand a maximum voltage of 14 V, namely the breakdown electric field is ~2.8 MV/cm.


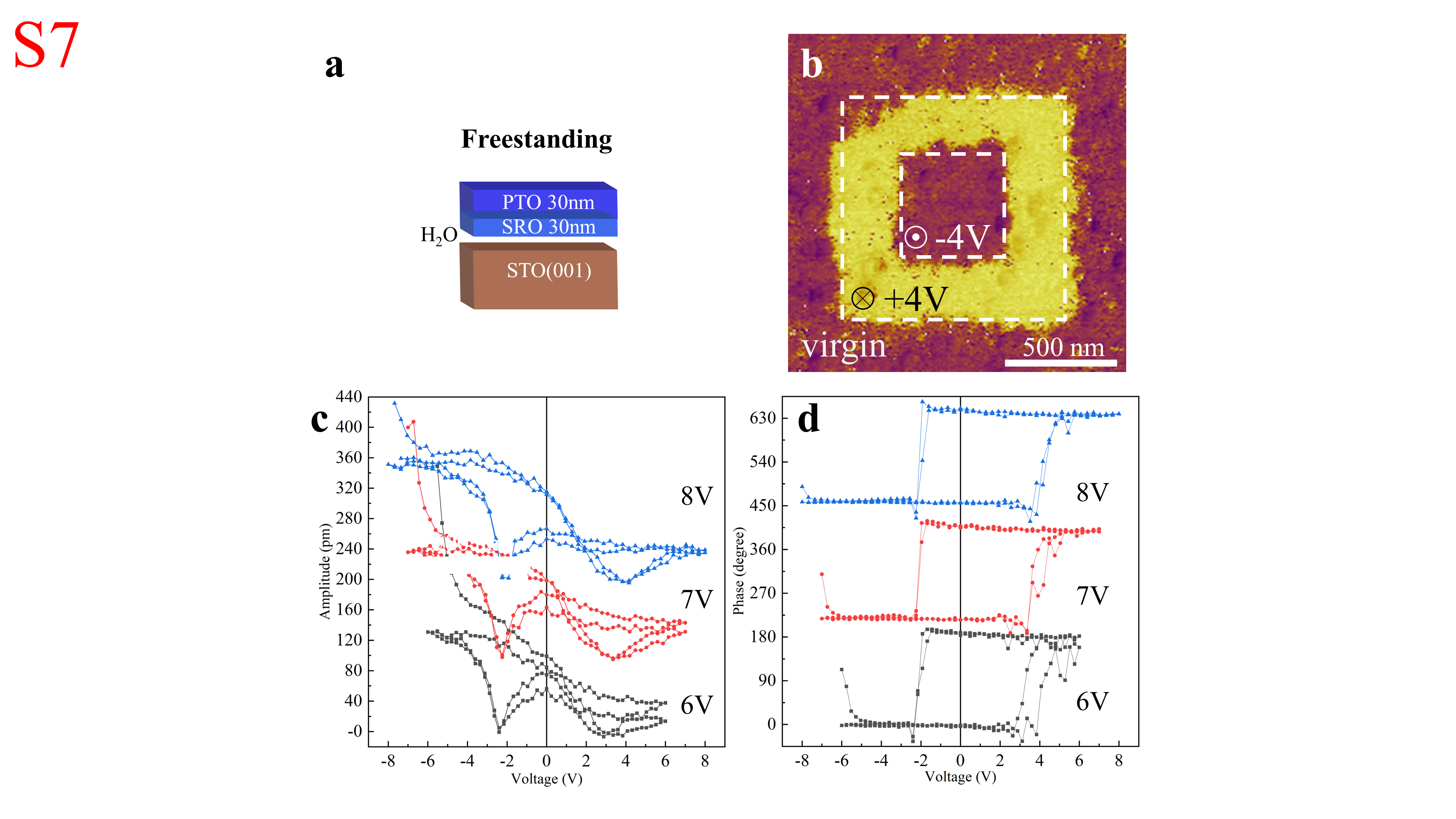


**Figure S7. Ferroelectricity of freestanding PTO (30 nm) / SRO (30 nm) bilayers.** a) Schematic structures of freestanding PTO/SRO bilayers. b) Out-of-plane PFM phase indicate its well-preserved ferroelectricity with scale bar is 500 nm. c,d) Amplitude-voltage and phase-voltage hysteresis loops show the freestanding bilayers can withstand a maximum voltage of 8 V, namely the breakdown electric field is ~2.7 MV/cm.

**3.** Large tensile strain Applied in Flexible Freestanding PTO Films


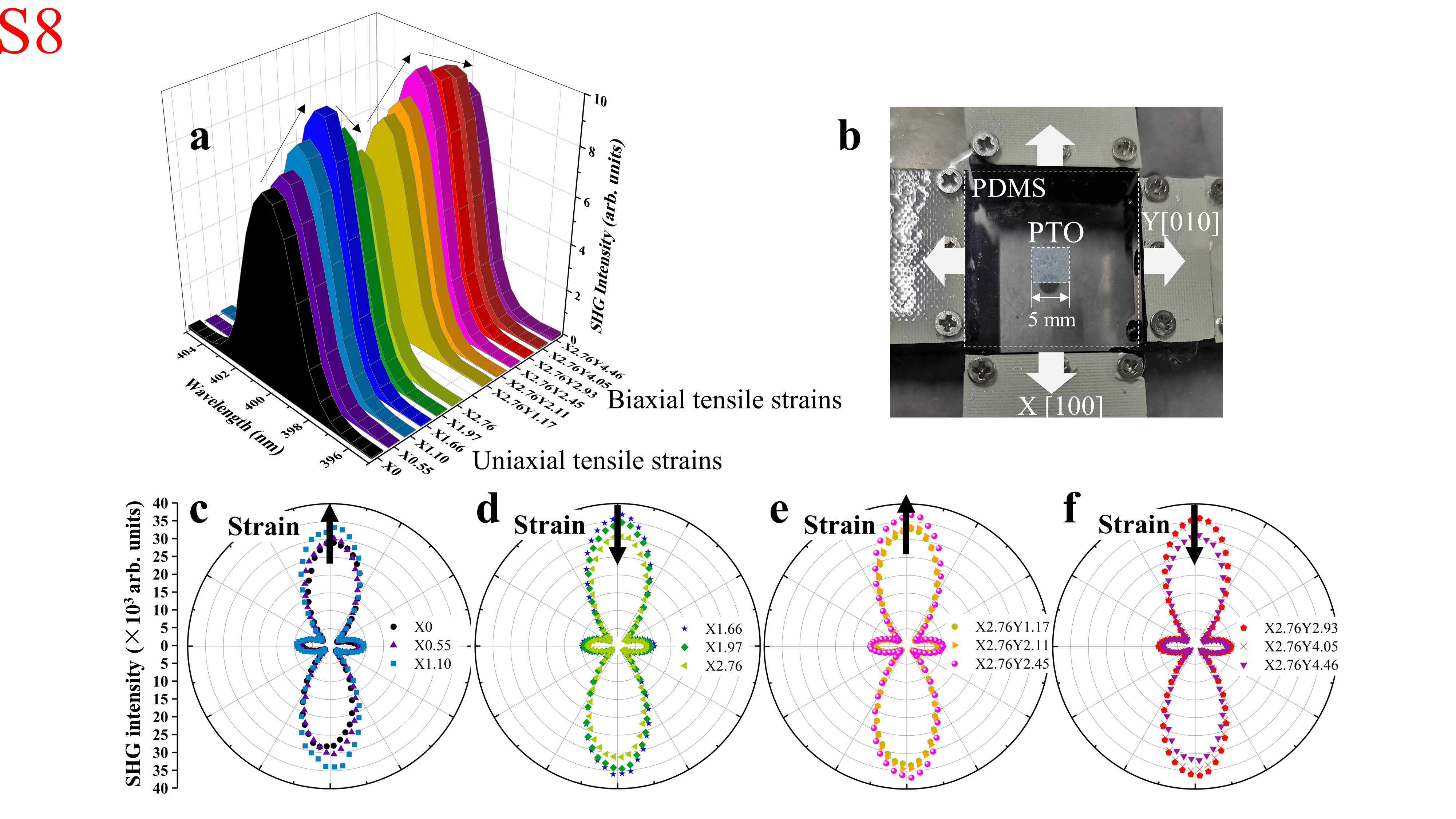


**Figure S8.** **SHG evolution for 50 nm-thick freestanding PTO films on polymer PDMS with increasing uniaxial and biaxial tensile strain.** a) SHG spectra of freestanding PTO films. b) Photo of 5 mm × 5 mm freestanding PTO films on flexible polymer PDMS substrates. c-f) SHG anisotropy polar plots measured in *p*-out configuration for freestanding PTO films with applying uniaxial tensile strain from 0% to 2.76% along *x* axis and applying biaxial tensile strain with 2.76% along *x* axis and up to 4.46% along *y* axis, respectively.


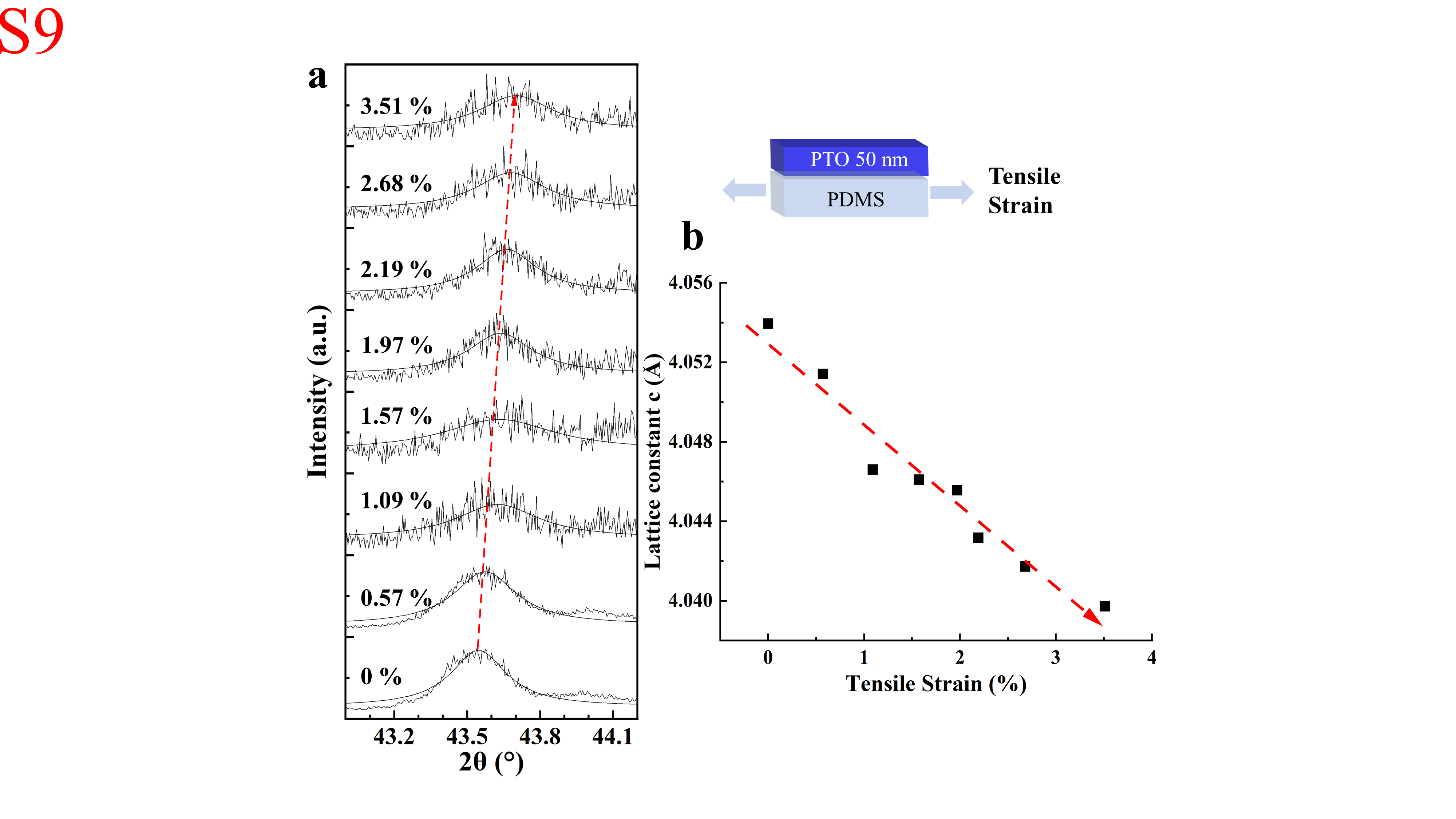


**Figure S9.** **X-ray diffraction intensity as a function of tensile strain in 50 nm-thick freestanding PTO films.** a) 2*θ*-*ω* scans around the PTO (002) diffraction peaks with increasing tensile strain (0%, 0.57%, 1.09%, 1.57%, 1.97%, 2.19%, 2.68%, and 3.51%). b) Lattice constant *c* with the tensile strain.

**4.** Structural and Ferroelectric Evolution of PTO Films at High Temperature


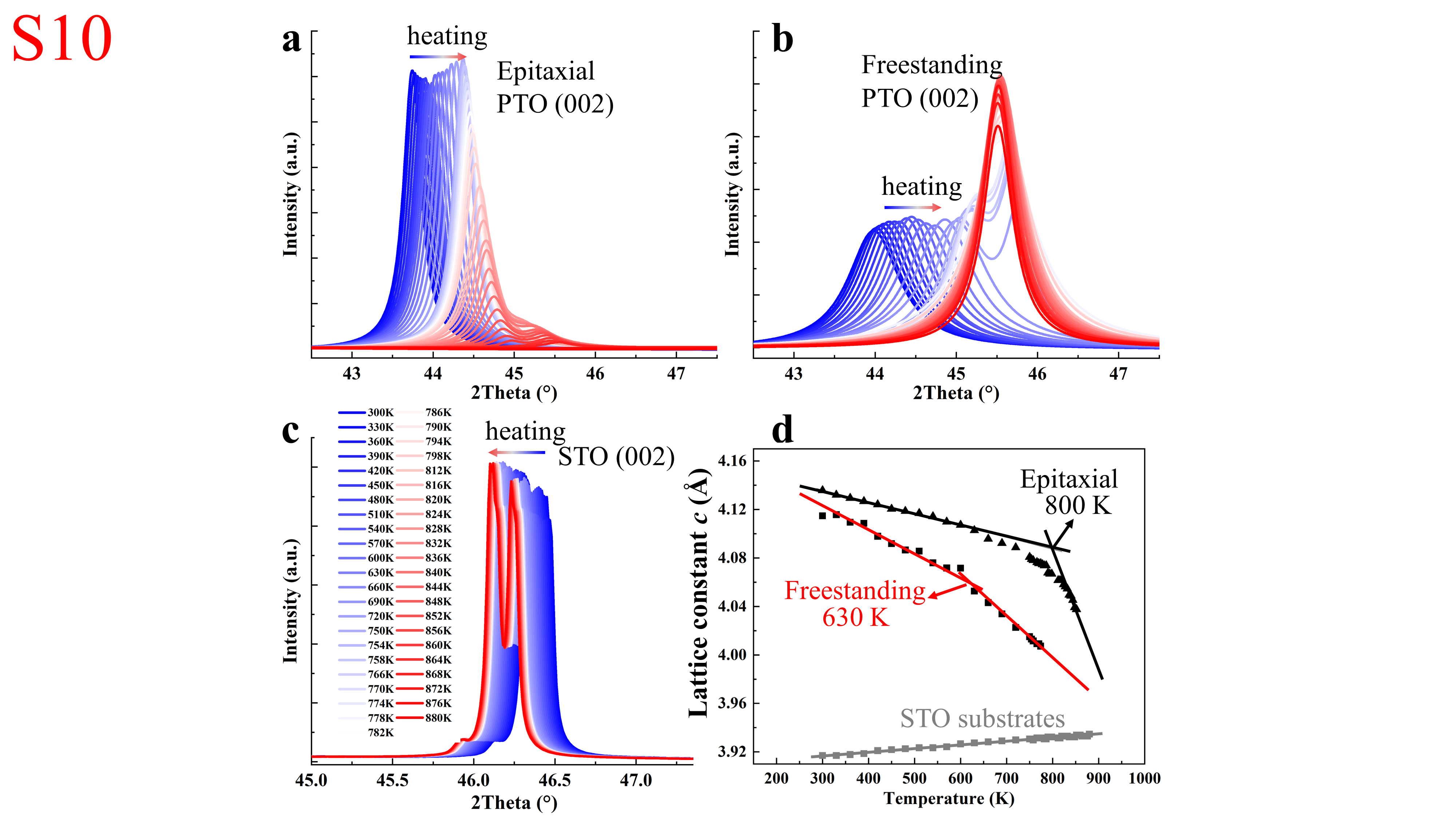


**Figure S10.** **XRD 2*θ*-*ω* scan of (00*l*) peaks from 300 K to 880 K**. a) Epitaxial PTO/STO films. b) Freestanding PTO films. c) (001)-orientated STO substrates. d) The out-of-plane lattice constants *c* decreased with increased temperature for both epitaxial and freestanding films, whereas increased with increased temperature for cubic STO substrates.


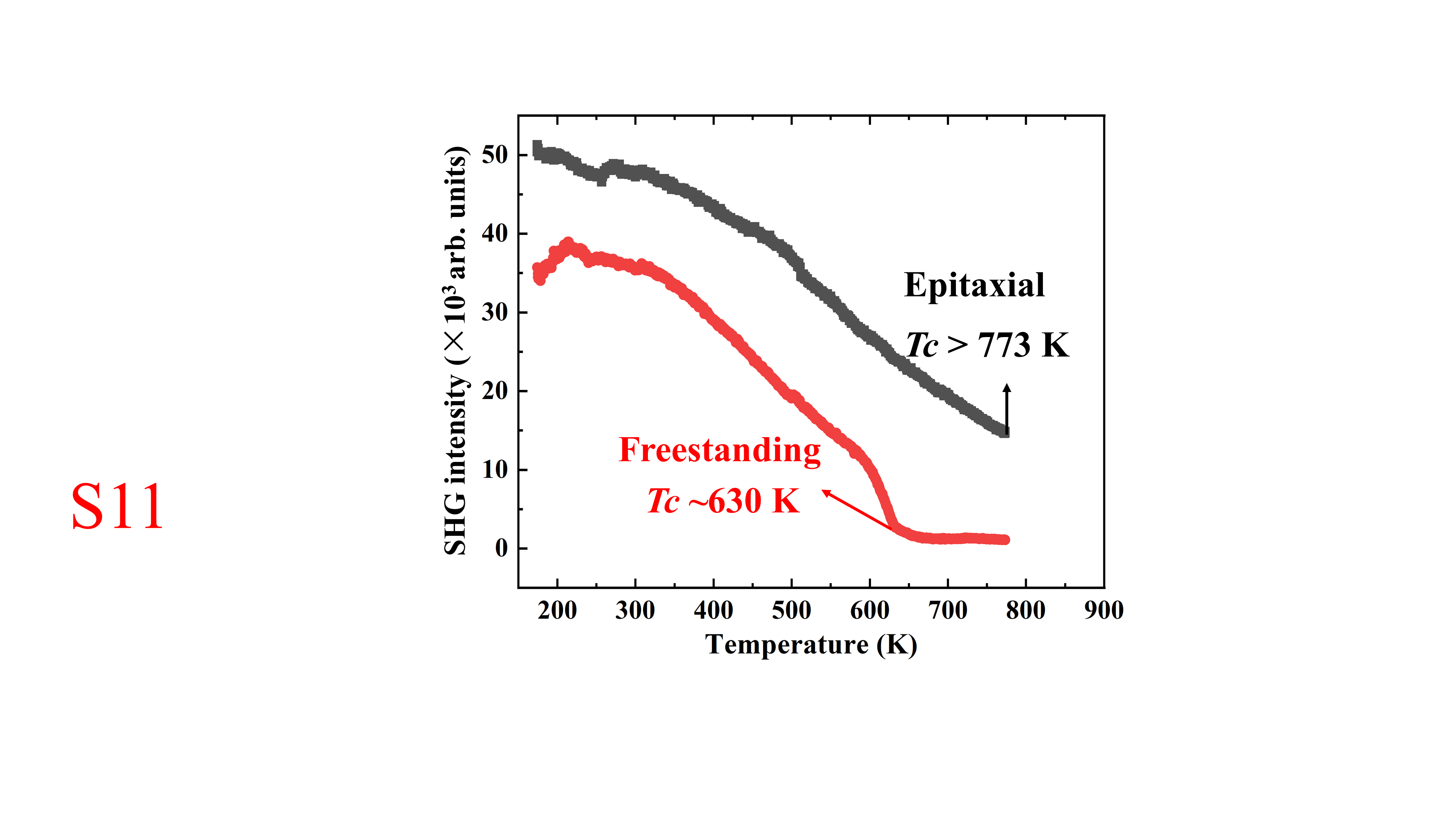


**Figure S11. SHG intensity vs temperature curves of epitaxial PTO/STO films and freestanding PTO films**. The Curie temperature *Tc* is above 773 K for epitaxial PTO/STO films and ~630 K for freestanding PTO films.

**5. Freestanding PTO films Transferred on Si wafers**

**
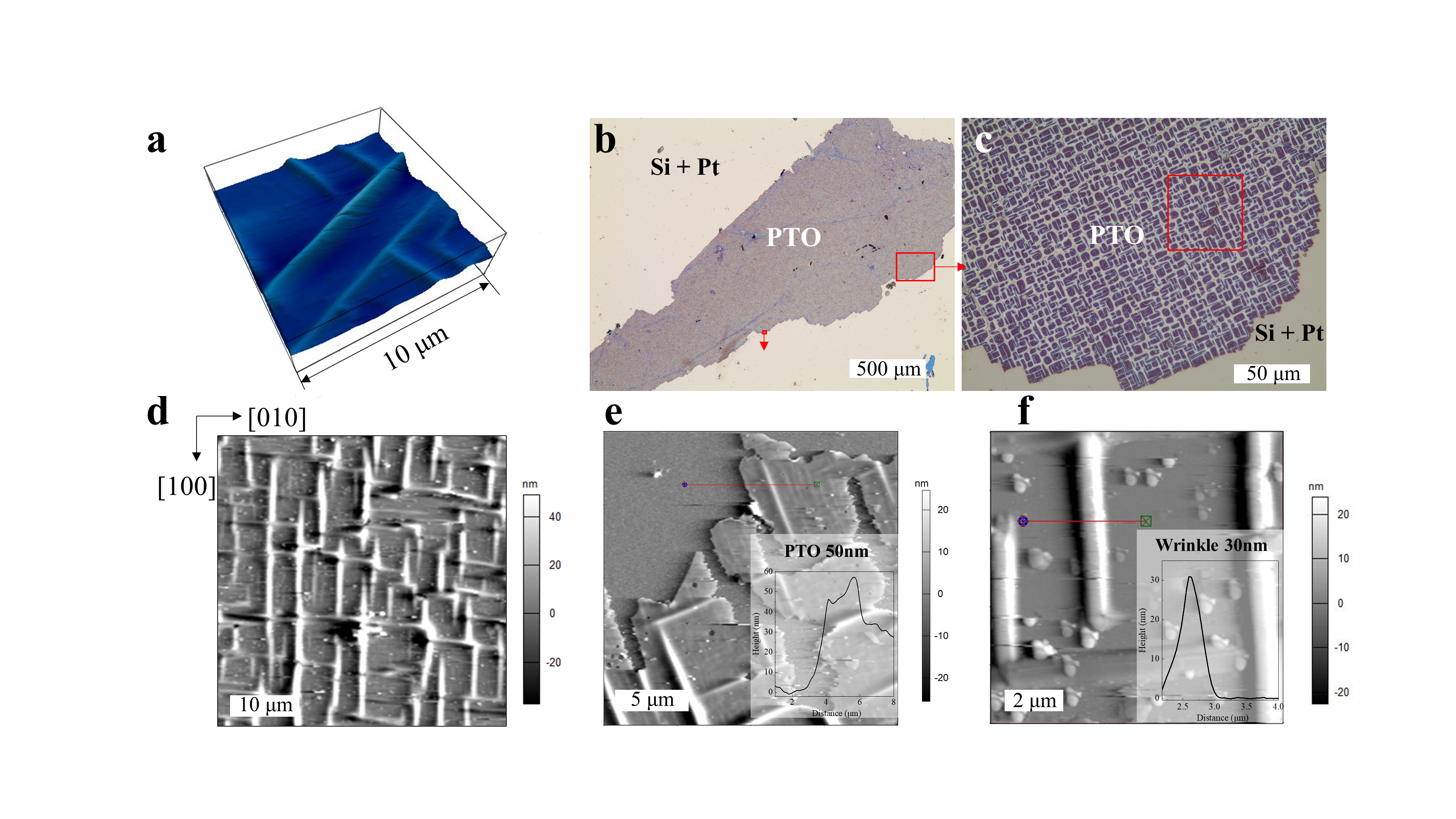
**

**Figure S12.** **Freestanding PTO nanosheets on Pt-covered Si wafers.** a) 3D image with wrinkles in 10 μm × 10 μm area. b,c) Light microscope images. d) AFM image which shows protrusions in wrinkles have formed along [100] and [010] in-plane orientation. e) AFM image which confirms the thickness of the PTO layer is about 50 nm. f) AFM image which shows that the protrusions of wrinkles have a thickness of ~30 nm.

[1] L. Zhang, J. Chen, L. Fan, O. Diéguez, J. Cao, Z. Pan, Y. Wang, J. Wang, M. Kim, S. Deng, J. Wang, H. Wang, J. Deng, R. Yu, J. F. Scott, X. Xing, *Science* **2018**, 361, 494.

[2] L. Han, Y. Fang, Y. Zhao, Y. Zang, Z. Gu, Y. Nie, X. Pan, *Adv. Mater. Interfaces* **2020**, 7, 1901604.

[3] D. Lu, D. J. Baek, S. S. Hong, L. F. Kourkoutis, Y. Hikita, H. Y. Hwang, *Nat. Mater.* **2016**, 15, 1255.

[4] H. Yao, K. Jin, Z. Yang, Q. Zhang, W. Ren, S. Xu, M. Yang, L. Gu, E.-J. Guo, C. Ge, C. Wang, X. Xu, D. Zhang, G. Yang, *Adv. Mater. Interfaces* **2021**, 8, 2101499.

[5] Jinfeng Zhang, Ting Lin, Liang Si, Ao Wang, Qingyu He, Huan Ye, Jingdi Lu, Qing Wang, Zhengguo Liang, Feng Jin, Shengru Chen, Minghui Fan, Er-Jia Guo, Qinghua Zhang, Lin Gu, Zhenlin Luo, Wenbin Wu, L. Wang, *Science* **2024**, 383, 388.

[6] Y. K. Wakabayashi, S. Kaneta-Takada, Y. Krockenberger, Y. Taniyasu, H. Yamamoto, *ACS Appl. Electron. Mater.* **2021**, 3, 2712.

[7] W. Zhou, W. Han, Y. Yang, L. Shu, Q. Luo, Y. Ji, C. Jin, Y. Zhang, J. Song, M. Ye, Q. Liu, S. Hu, L. Chen, *Appl. Phys. Lett.* **2023**, 122, 062901.

[8] G. Dong, S. Li, T. Li, H. Wu, T. Nan, X. Wang, H. Liu, Y. Cheng, Y. Zhou, W. Qu, Y. Zhao, B. Peng, Z. Wang, Z. Hu, Z. Luo, W. Ren, S. J. Pennycook, J. Li, J. Sun, Z.-G. Ye, Z. Jiang, Z. Zhou, X. Ding, T. Min, M. Liu, *Adv. Mater.* **2020**, 32, 2004477.
